# The relationship of prenatal antibiotic exposure and infant antibiotic administration with childhood allergies: a systematic review

**DOI:** 10.1186/s12887-020-02042-8

**Published:** 2020-06-27

**Authors:** Ruth Baron, Meron Taye, Isolde Besseling-van der Vaart, Joanne Ujčič-Voortman, Hania Szajewska, Jacob C. Seidell, Arnoud Verhoeff

**Affiliations:** 1 Sarphati Amsterdam, Nieuwe Achtergracht 100, 1018 WT Amsterdam, the Netherlands; 2 Winclove Probiotics, Hulstweg 11, 1032 LB Amsterdam, the Netherlands; 3 Department of Paediatrics, Medical University of Warsaw, Warsaw, Poland; 4 Department of Health Sciences, Amsterdam Public Health research institute, Vrije Universiteit Amsterdam, De Boelelaan 1085, 1081 HV Amsterdam, the Netherlands

**Keywords:** ‘Antibiotic exposure’, Pregnancy, ‘Childhood allergies’, Asthma, Eczema, ‘Hay fever’, Microbiome

## Abstract

**Background:**

Early antibiotic exposure may be contributing to the onset of childhood allergies. The main objective of this study was to conduct a systematic review on the relationship between early life antibiotic exposure and childhood asthma, eczema and hay fever.

**Methods:**

Pubmed and Embase were searched for studies published between 01-01-2008 and 01-08-2018, examining the effects of (1) prenatal antibiotic exposure and (2) infant antibiotic administration (during the first 2 years of life) on childhood asthma, eczema and hay fever from 0 to 18 years of age. These publications were assessed using the Newcastle Ottawa Scale (NOS) and analysed narratively.

**Results:**

(1) **Prenatal antibiotics**: *Asthma* (12 studies): The majority of studies (9/12) reported significant relationships (range OR 1.13 (1.02–1.24) to OR 3.19 (1.52–6.67)). Three studies reported inconsistent findings. *Eczema* (3 studies): An overall significant effect was reported in one study and in two other studies only when prenatal antibiotic exposure was prolonged. (2) **Infant antibiotics**: *Asthma* (27 studies): 17/27 studies reported overall significant findings (range HR 1.12 (1.08–1.16) to OR 3.21 (1.89–5.45)). Dose-response effects and stronger effects with broad-spectrum antibiotic were often reported. 10/27 studies reported inconsistent findings depending on certain conditions and types of analyses. Of 19 studies addressing reverse causation or confounding by indication at least somewhat, 11 reported overall significant effects. *Eczema* (15 studies): 6/15 studies reported overall significant effects; 9 studies had either insignificant or inconsistent findings. *Hay fever* (9 studies): 6/9 reported significant effects, and the other three insignificant or inconsistent findings. **General:** Multiple and broad-spectrum antibiotics were more strongly associated with allergies. The majority of studies scored a 6 or 7 out of 9 based on the NOS, indicating they generally had a medium risk of bias. Although most studies showed significant findings between early antibiotic exposure and asthma, the actual effects are still unclear as intrapartum antibiotic administration, familial factors and confounding by maternal and child infections were often not addressed.

**Conclusions:**

This review points to a moderate amount of evidence for a relationship between early life antibiotics (especially prenatal) and childhood asthma, some evidence for a relationship with hay fever and less convincing evidence for a relationship with eczema. More studies are still needed addressing intra-partum antibiotics, familial factors, and possible confounding by maternal and childhood infections. Children exposed to multiple, broad-spectrum antibiotics early in life appear to have a greater risk of allergies, especially asthma; these effects should be investigated further.

## Background

Childhood allergies are rising in prevalence around the world, with more rapid increases occurring in low and middle income countries, as these countries become more affluent [1]. It is estimated that worldwide 14% of children have asthma [2] and 7.9% have eczema [3]. Estimations for allergic rhinitis (hay fever) worldwide are 20.7% in 6-7 year olds and 33.2% in 13-14 year olds [4].

Asthma, eczema and allergic rhinitis are chronic inflammatory disorders of the lung, skin and nasal mucous membrane, respectively [5, 6]. Besides the discomfort experienced with these allergies, such as the shortness of breath and chest tightness typical of asthma, other co-morbidities including ear infections, sinusitis, sleeping disorders, overweight, pain, itching, emotional problems and cognitive disorders can contribute to a detrimental quality of life [7, 8]. The costs of chronic allergies are substantial for society due to medical costs, parental absence at work and children missing school days [7, 9].

Asthma and other allergies are considered to develop through a combination of genetic and environmental factors [6]. Besides familial allergies, other known risk factors for asthma are maternal smoking, delivery mode, childhood infections, diet, pollutants in the environment and antibiotic usage; breastfeeding and sufficient maternal vitamin D levels are considered to be protective [10]. Human and animal studies have shown that disruptions to the gut microbiome in early life may influence the development of chronic health conditions, such as allergies [11]. The microbiota has a wide range of functions including protection from pathological bacteria, the synthesis of vitamins (eg. vitamins K, B12 and other B vitamins), contributing to energy metabolism and the absorption of nutrients, shaping the immune system by forming lymphatic structures and differentiating lymphocytes, such as T cells and B cells, and guiding neurological development [12, 13].

The first six months after birth is a period of rapid microbiome development and this period is considered to be a time of susceptibility to long term changes to the microbiome [14]. Of all disrupting factors, early antibiotic exposure is considered to have the greatest impact on the gut microbiome in infants [15], leading to a disturbed microbiome still months and sometimes years after antibiotic treatment [16]. Collateral damage caused by antibiotics can entail the loss of important bacteria and a reduced diversity of bacteria, which in turn can lead to the growth of pathogens, to changes in metabolic processes and to an impaired immune system [14]. Even if the gut finally regains its diversity after antibiotic treatment, the bacterial composition may already have changed permanently [17].

The effects of early antibiotic exposure may already begin during pregnancy. Various human and animal studies have shown that maternal antibiotic administration during pregnancy and during delivery can modify the gut microbiome of the infant [18, 19]. Antibiotic use during pregnancy and delivery is common. A Dutch study found that during the period 1994-2009, 20.8 % of pregnant women had received antibiotics by 39 weeks of pregnancy [20]. This proportion is likely to have been much higher if antibiotic administration during delivery had also been included. A Danish study found that at least 41.5% of women had received antibiotics during pregnancy, including intra-partum antibiotics [21]. The most common reasons to prescribe antibiotics during pregnancy are for urinary tract infections and respiratory diseases. The main reasons for prescribing antibiotics just prior to and during delivery are to prevent group B Streptococcus infection in the newborn and to prevent other infant and maternal infections associated with preterm birth, epidurals and caesarean sections [22].

The administration of antibiotics to infants is also very common. In high income countries more than half of all infants have had antibiotic treatments during their first months of life [15]. Common reasons for prescribing antibiotics to children from 0-2 years of age are for Otitis Media Acuta (OMA), followed by acute upper respiratory tract infections (URTI) and fever [23]. Most antibiotics that are prescribed are broad-spectrum antibiotics (such as amoxicillin, macrolides, betalactams and cephalosporins) which work against a wide range of diseases, but can cause a lot of damage to the microbiome. Dekker et al.2017 [23] found that of all antibiotic prescriptions for children in the Netherlands aged 0-2 years, 72% were for amoxicillin, followed by 13% for macrolides.

As antibiotics are so commonly administered to pregnant women and children, it is important to understand the extent that they may inadvertently be contributing to the onset of chronic diseases, such as allergies. The aim of this study is to summarize and evaluate the evidence obtained from studies published over the last 10 years (2008 to 2018) regarding the relationship of prenatal (conception till birth) antibiotic exposure and infant (0-2 years) antibiotic administration with childhood allergies, focusing on asthma, eczema and hay fever.

## Methods

### Inclusion criteria

The inclusion criteria for this review were human subjects, observational studies written in English and examining the relationship of any exposure to antibiotics during pregnancy and early life with childhood allergies (asthma, eczema or hay fever) from 0-18 years of age, effect sizes (e.g. odds ratios (OR), hazard ratios (HR) and relative risks (RR)) and confidence intervals were reported and multivariable analyses had been conducted. As previous systematic reviews on one or more of these allergies have covered publications up till several years ago [24–28], we chose to summarize the newest evidence available by focusing on the last 10 years (published in any scientific journal from 01-01-2008 until 01-08-2018). The protocol of this study is available at the PROSPERO international prospective register of systematic reviews, with registration number CRD42019126447.

### Exposure and outcome variables

The exposure of this study was the administration of any type of prenatal antibiotic throughout pregnancy including delivery and during early childhood up to two years of age. Data could be collected from medical records, prescription databases or by maternal self-report. The allergies examined were eczema, hay fever and asthma during childhood (0-18 years). Although some types of asthma are not triggered by allergens (non-allergic asthma), the vast majority of asthma cases in childhood are considered to be of the allergic type [29]. In this review, we therefore refer to childhood asthma as being an allergy. Although wheezing may be an early symptom of asthma, we excluded wheezing for this review. About half of all children will experience some transient wheezing, most of whom will not go on to develop asthma [30]. Data documenting these allergic conditions could be retrieved from medical records, prescription databases, parental report, or from a doctor’s diagnosis. These sources of data were documented so that they could be taken into consideration when evaluating the articles.

### Search strategy

An extensive search was conducted in the databases Pubmed and Embase (supplementary figure S1). The references of publications were also screened for relevant literature. The titles and abstracts were initially screened for relevance to the current study by RB. The full text of each potentially relevant publication was then independently read by two researchers (RB and MT) to determine its eligibility for the study. Any discord between the researchers regarding selection was discussed to reach consensus. Reasons for exclusion from the current study were documented.

### Evaluation of articles for quality and risk of bias

The final publications deemed suitable for inclusion were then evaluated independently by RB and MT for their risk of bias, using the Newcastle Ottawa Scale (NOS) as a guide for cohort studies and case-control studies [31]. The NOS divides the assessment into three main categories; for cohort studies the following categories apply: 1) ‘Selection’ assesses the representativeness of the study population, objectivity of the exposure measurements and evidence that the outcome was not already present at the start of the study; 2) ‘Comparability’ examines whether relevant confounders have been adequately accounted for; 3) ‘Outcome’ covers the objectivity of outcome measurements, adequacy of follow-up time and risk of bias due to loss to follow-up. For each study the different categories were awarded points if they had been addressed adequately. These points were added up to obtain a score, the maximum being 9 points, signifying the lowest risk of bias. This evaluation was categorized into low risk of bias (8-9 points) medium risk of bias (6-7 points) and unclear risk of bias (<6 points).

The assessment for case-control studies also covers the adequacy of case definition, the certainty of there being no history of the outcome in the control group and non-response rates in cases and controls.

Common issues encountered in studies examining the relationship between antibiotics and childhood allergies are reverse causation and confounding by indication [32]. Reverse causation occurs when early symptoms of the outcome, such as asthma, are already present during the exposure period, leading to the administration of antibiotics. Confounding by indication occurs when antibiotics are given for infections, such as respiratory infections, which are risk factors for childhood asthma [33]. As these important aspects are not reflected in the NOS score, we examined each publication covering infant antibiotic administration for these potential issues and reported these separately.

### Data extraction

RB and MT also independently extracted relevant characteristics and data from each of the publications. This information included the type of study, country, sample size, exposure and outcome measurements, prevalences of exposure and outcome and the effect sizes of the main analyses. Potential confounders and other influential factors were also examined in each publication. These were the dosage, timing and types of antibiotics, gender and birth weight of the infant, delivery mode, breastfeeding, familial allergies, maternal and childhood infections, prenatal antibiotic exposure (for studies on infant antibiotic administration) and infant antibiotic administration (for studies on prenatal antibiotic exposure). The evaluations and data extractions carried out by RB and MT were subsequently compared and any discord was discussed till consensus was reached. The import and storage of articles, screening, selection, evaluations and data extractions were carried out using the systematic review assistance software Covidence (www.covidence.org). An initial assessment of all the included studies revealed there was much heterogeneity within the different study topics, with regard to the age and dosage of antibiotic exposure, follow-up times, age and type of measurements of outcomes and the numbers and types of confounding factors that were accounted for. Therefore, the authors concluded that a narrative synthesis would be more appropriate than a meta-analysis.

## Results

Based on the search terms and filters, Pubmed yielded 1198 publication titles and Embase 3725 publications (supplementary figure S1). No further publications were identified from the reference lists examined. After removing the duplicates, 4046 titles/abstracts remained for screening. Seventy-four full texts of publications were read and assessed for eligibility for this review, of which 48 publications were finally selected for inclusion. The reasons for exclusion after full text screening are reported in the supplementary data flowchart S1.

### Study findings

In total, 48 publications were identified examining the relationship of prenatal antibiotic exposure and infant antibiotic administration with childhood allergies, five of these examining both prenatal as well as infant antibiotics. Twelve publications investigated the relationship between prenatal antibiotic exposure and asthma [34–45]; three publications investigated prenatal antibiotic exposure and eczema [46–48]. No publications were identified examining prenatal antibiotic exposure and hay fever. Of the publications examining infant antibiotic administration, 27 publications investigated asthma [35, 42, 43, 45, 49–71], 15 publications investigated eczema [48, 64–77] and nine publications investigated hay fever [64–68, 78–81], eight of these publications examining more than one of these allergies. Studies varied with respect to their objectives. Some aimed to estimate the relationship between antibiotics and childhood allergies, taking into account various potential confounders and others aimed to identify significant predictors of childhood allergies from a range of possible factors, including antibiotic exposure.

### Prenatal antibiotic exposure and childhood asthma

#### Study characteristics

The 12 studies examining prenatal antibiotic exposure and childhood asthma were conducted in the United States (n=3), Denmark (n=2), Canada (n=2), Japan (n=1), Iran (n=1), the Netherlands (n=1), Sweden (n=1) and Finland (n=1) (supplementary table S2a). Eight were cohort studies and four were case-control studies. Two of the cohort studies and one case-control study conducted additional sibling-matched analyses.

The sample sizes ranged from 134 case-control pairs to 910,301 children. The prevalence of prenatal antibiotic exposure in the studies ranged from 20% - 36%; however, two studies reported that they had excluded intra-partum antibiotics and for the remaining 10 studies, it was unclear whether intra-partum antibiotics had been included as part of the exposure. Estimates of childhood asthma in the cohort studies ranged from 6% - 14.8%. The children’s age of outcome ranged from 0-5 years in one study till 7-14 years in another study.

#### Main findings

All studies, except for one sub-study showed a positive trend in the relationship between prenatal antibiotics and childhood asthma, of which the majority were significant, ranging from OR 1.13 (1.02-1.24) to OR 3.19 (1.52- 6.67). Insignificant effect sizes ranged from HR 0.99 (0.92-1.07) (Sibling-matched analysis) to HR 1.17 (1.00-1.32). Two studies by Loewen et al.(2018) and Stockholm et al. (2014) reported a significant association, but found that this increased risk of childhood asthma was not only limited to antibiotic exposure during pregnancy [34, 41]. Childhood asthma was also significantly associated with maternal antibiotic usage during the periods before and after pregnancy and showed similar effect sizes. Two other studies by Mulder et al., (2016) and Ortqvist et al.,(2014) found a significant relationship in their main population analyses, but after conducting additional case-sibling analyses, this relationship lost its significance [37, 43]. Another study conducting an additional sibling-matched cohort study found an even greater significant effect in the sibling study [35]. This study had only stratified for gender and antibiotic types but had not taken other potential confounders into account.

#### Influential factors

##### Confounding by indication and by other factors

The majority of studies examined or adjusted for other influential factors in the relationship between prenatal antibiotics usage and childhood asthma. Potential confounders generally considered were maternal and/or familial asthma, infant gender, maternal age, ethnicity, education, smoking during pregnancy, parity and birth weight. Relevant potential confounders often not taken into account were delivery mode, maternal infections, breastfeeding and postnatal child antibiotic usage. Confounding by indication (such as by maternal respiratory infections) was addressed somewhat in four studies by additional examination of the types of antibiotics generally used for different infections, such as respiratory and urinary tract infections. Two studies found that antibiotics used to treat maternal respiratory infections had a stronger effect than antibiotics used to treat maternal urinary tract infections, although the effects of both types of antibiotics were still significant [41, 43]. Metsala et al. (2014) found the strongest association for antibiotics treating both respiratory diseases and urinary tract infections, but no significant effect for antibiotics only treating urinary tract infections [42]. These studies suggested that there may have been at least some confounding by maternal respiratory tract infections. Stensballe et al. (2013) found the opposite result with maternal antibiotics used to treat non-respiratory diseases having a significant and stronger effect than mothers using any types of antibiotics, suggesting a causal role of antibiotics [44].

The majority of studies did not examine postnatal antibiotic administration as a possible confounder or mediator (8/12). One study mentioned purposely not adjusting for postnatal antibiotics, so as not to underestimate the effect of prenatal antibiotics [37]. Loewen et al., (2018) [34] corrected for postnatal antibiotic use up to 12 months after birth and in three other studies, postnatal antibiotics was found to be an independent predictor of asthma, besides prenatal antibiotic exposure [38, 40, 45]. Lapin et al., (2015) [40] showed that prenatal antibiotic exposure was still significantly associated with asthma, after excluding children who had taken antibiotics for early respiratory infections.

##### Antibiotics: dose, type and timing

The studies examining any evidence for a dose-response relationship found that each additional course or prescription for antibiotics was associated with an increased risk for asthma. The most commonly mentioned antibiotics with significant associations were cephalosporins [35, 42], extended-spectrum penicillins [34, 37, 42], sulphonamides and trimethoprim [34, 37, 42] and macrolides [42]. There were inconsistent findings with regard to the timing of prenatal antibiotics usage during pregnancy. Adding to the complication of the effects of the timing of exposure, there was unclarity in most studies about whether or not they had included intra-partum antibiotics as part of the exposure.

##### Outcome: Age of onset

Three studies examined the effects of antibiotics at different age groups of asthma onset [35, 41, 42]. All studies showed a stronger effect of antibiotics at earlier ages (e.g. Metsala, 2014: 3-5 years (OR 1.32) versus 6-9 years (OR 1.23): both significant; Yoshida, 2018: 1-3 years (HR 1.18) significant versus 3-6 years (HR 1.09) (insignificant)) [35, 42].

### Prenatal antibiotic exposure and childhood eczema

#### Study characteristics

Three studies were identified examining prenatal antibiotic exposure and childhood eczema and were conducted in Denmark, United States and Belgium (supplementary table S2b). All were cohort studies. One study solely focused on the effects of intra-partum antibiotics [47] and the other two studies [46, 48] did not mention whether intra-partum antibiotics were included. The prevalence of eczema in these studies ranged from 16% at 18 months to 36.3% up to 4 years of age.

#### Main findings and influential factors

One of the three studies found a significant relationship between prenatal antibiotic exposure and eczema (OR 1.82 (1.14-2.92): Dom et al., 2010) [48] and the other two found significant relationships only under certain conditions, such as intra-partum exposure for more than 24 h (Wohl et al., 2015) [47]and the child’s mother having atopy, as well as antibiotic exposure in the 1^st^ or 2^nd^ and 3^rd^ trimester (Timm et al., 2016) [46]. Timm er al. found that for children who had additionally been born by caesarean section, the significant effect size was even stronger. Dose-response relationships were not examined. The two studies examining antibiotic type did not observe any differences with regard to antibiotic type and eczema [46, 47]. The study examining intra-partum antibiotics found no differences in eczema between children with and without family members with allergies [47].

### Infant antibiotic administration and childhood asthma

#### Study characteristics

Twenty seven studies (supplementary tables S3a and S3d) investigated the relationship between infant antibiotic administration and childhood asthma; these were conducted in Sweden (5), Canada (3), United States (4), Taiwan (1), Japan (2), New Zealand (2), United Kingdom (1), Colombia (1), Poland (1), the Netherlands/Scotland (1), Portugal (1), South Korea (1), Iran (1), Italy (1), Finland (1) and Australia (1). Seventeen were cohort studies, five were cross-sectional studies, three were case-control studies and two contained two sub-studies with different designs: one prospective and one case-control (sibling-matched). The sample sizes ranged from 198 to 792,130 children in the studies examining asthma. The age of asthma outcome ranged from 0-4 years till 13-14 years.

The prevalence of infant antibiotic administration ranged from 4.6% in the first week of life to 14.1% in the first three months following birth. Antibiotic administration during the first 6 months ranged from 16% to 33.1% and antibiotic administration reported during the 1st year of life ranged from 23.1% to 87%. The most commonly reported prevalences of asthma ranged between 6% and 12%, with outliers of 4.4% and 28.8%.

#### Main findings

Over half of all publications (17/27) reported there was an overall significant relationship between infant antibiotic administration and childhood asthma ranging from HR 1.12 (1.08-1.16) to OR 3.21 (1.89-5.45). Further analyses, of these populations revealed at times that the significant relationship was often driven by certain subgroups of children, such as those without ear infections, those with asthma onset before preschool age, or only those who had been administered cephems [51, 56]. One study by Almqvist (2012) [57] reported a significant relationship, but concluded this may be due to reverse causation or confounding by infection, as the significance was driven by antibiotics used to treat respiratory tract infections and not by antibiotics for urinary tract or skin infections. Yoshida et al., (2018) [35] also found a significant relationship between early antibiotics and asthma after conducting an additional sibling study designed to take familial characteristics into account. This study did not take any confounding by indication into account, however.

Another 10 studies reported either overall insignificant findings or both significant as well as insignificant relationships depending on certain conditions and types of analyses. Reasons for these inconsistent findings included significant effects becoming lower or insignificant after additional analyses, such as adjusting for respiratory infections [59, 82], or number of physician visits [61] or after conducting sibling-matched sub-studies [43], or after excluding children with any wheezing from the exposure period [67]. Kusel et al., (2008) found an insignificant relationship after adjusting for number of GP visits and antibiotic propensity score (probability estimation of having received antibiotics for each infection in the first year of life) [71]. Wang et al., (2013) conducted two sub-studies from different periods of time (1998 and 2003) and only found a significant relationship in one sub-study (1998) [66]. Mai et al., (2010) containing two sub-studies with different ages of outcome (4 years and 8 years) only found significance in the study with 4 years of age as outcome [68].

#### Influential factors

##### Confounding by indication and by other factors

Potential confounders often taken into account were maternal or familial asthma/allergies, infant gender, maternal age, ethnicity, education, smoking in home, parity and birth weight. Important potential confounders not always taken into account were infectious diseases (considered at least somewhat in 16/27 publications), delivery mode (considered in 14/27 publications) and prenatal antibiotics (considered in 6/27 publications).

Nineteen publications addressed reverse causation and/or confounding by indication at least to some degree; findings in these 19 publications ranged from effect size OR 0.78 (0.46-1.32) to OR 2.3 (1.2-4.2). Eleven of these still reported significant associations after having adjusted for one or more infectious diseases or having taken reverse causation into account.

##### Antibiotics: dose, type and timing

A dose-response relationship was found in the majority of studies that had examined this. Broad-spectrum antibiotics were found to have stronger effects than narrow-spectrum antibiotics [57, 70]. Macrolides, cephalosporins and amoxicillin were most frequently mentioned as having the strongest effects [35, 42, 53, 57, 59, 62]. In the few studies examining antibiotic exposure at various ages, two studies reported antibiotics as having a stronger effect when the exposure was in the 1^st^ year compared to the 2^nd^ year [43, 49]. One study, however, showed that only antibiotic exposure after 15 months (versus before 15 months) had a significant effect [67] .

##### Asthma: age of onset

In the eight studies that examined different age groups of asthma onset, there was always a stronger effect of antibiotics at younger ages of asthma onset [35, 42, 43, 53, 56, 57, 68, 70]. Metsala et al., (2014) [42], for example, reported significant odds ratios of 1.68 at 3-5 years versus 1.33 at 6-9 years and Yoshida et al.(2018) [35] reported significant hazard ratios of 2.43 at 1-2 years versus 1.23 at 3-5 years. Almqvist et al. (2012) [57] reported a significant effect at 1-2 years, but insignificant effect at 3+ years and Goksor et al. (2013) [56] reported a significant effect when the age of onset was before preschool age and an insignificant effect from preschool age onwards.

### Infant antibiotic administration and childhood eczema

#### Study characteristics

The 15 publications (supplementary tables S3b and S3d) examining eczema as outcome were conducted in the United Kingdom (2), New Zealand (2), Australia (1), Netherlands (1), Singapore (1), Spain (1), Sweden (1), Belgium (1), Germany (1), United States (1), Japan (1), South Korea (1) and Taiwan (1). Twelve studies were cohort studies and the other three cross-sectional studies. The sample sizes ranged from 198 to 792,130 children in the studies examining eczema. The age of eczema outcome ranged from 0-1 years till 8 years of age.

The prevalence of antibiotic usage in these publications ranged from 16% in the first 6 months to 67.5% in the first year of life. The prevalence of eczema also varied from 16% at 8 years of age to 39% at 15 months of age.

#### Main findings

Six out of the 15 publications reported significant relationships between infant antibiotic administration and eczema. Significant OR effect sizes ranged from OR 1.20 (1.02-1.41) to OR 3.11 (1.10-8.76) and significant HR effects sizes ranged from HR 1.18 (1.16-1.19) to HR 1.61 (1.53-1.70). Five publications concluded that there was no relationship between infant antibiotics and eczema, with effect sizes ranging from OR 0.61 (0.36-1.01) to OR 1.5(0.8-3.6). Four more publications had inconsistent findings: one showed the relationship to be significant only at a later age of eczema onset (12-18 months versus 6-12 months) [75] ; another publication with two sub-studies of children born in 1998 and 2003 respectively, only found a significant relationship in 1998 [66]. One study showed that antibiotic administration before three months of age was not significantly associated with eczema from 3-12 months, but the authors suggested that antibiotic administration before 15 months may be associated with eczema at 4 years of age [70]. This relationship with eczema remained significant after adjusting for chest infections, but lost its significance after adjusting for other factors, such as family history of allergies . Schmitt et al., (2010) [77] showed that the relationship between any antibiotic administration during the first year and eczema in the second year was insignificant, but became significant when children had had at least two antibiotic courses.

#### Influential factors

##### Confounding by indication and by other factors

Most studies took a wide range of potential confounders into account, such as family history of allergies, gender, birth weight, smoking, pets and number of siblings. Relevant potential confounders not always considered were infectious diseases (examined somewhat in 6/15 studies), delivery mode (7/15 studies) and prenatal antibiotics (examined in 2/15 studies). Of the eight studies that had taken confounding by indication or reverse causation at least somewhat into account, two showed significant relationships [65, 67]. One study that conducted sub-studies of two cohorts of 2-6 year olds born in 1998 and 2003, only found a significant effect in 1998 [66].

One study found that the effect of antibiotics on eczema was only significant in children without diagnosed respiratory tract infections [66]. A subgroup analysis in another study revealed that the effect of antibiotic usage on eczema was stronger in a sample of children who concurrently had asthma or rhinitis, than in children without asthma or rhinitis [76].

##### Antibiotics: dose, type and timing

Of the six publications examining whether there was a dose-response relationship, one reported a dose –response relationship (Schmitt, 2009) [77] and one found a dose-response relationship in one of two cohorts examined (1998 cohort, but not 2003 cohort; Wang,2013) [66]. Most studies did not examine antibiotic types, but Yamamoto-Hanada, (2017) [65] found that the significant relationship with eczema was mainly driven by macrolides. Schmitt et al. (2010) found that infections of the respiratory tract during the first year of life were protective, but insignificant; however, respiratory tract infections treated with macrolides or cephalosporins were significant risk factors for eczema in the second year of life. The effects of the timing of antibiotic exposure were generally not examined. Dom et al.,(2010) [48] found, however, that although prenatal antibiotic exposure was a significant risk factor for eczema, childhood antibiotic usage during the first year was protective but insignificant, and childhood antibiotic usage after the first year was significantly protective for eczema.

##### Eczema: age of onset

The majority of studies did not compare different childhood ages of eczema onset. One study found that earlier age of eczema onset (6-12 months) was mainly associated with familial factors, such as maternal allergic history and not with antibiotic usage, while later onset eczema (12-18 months) was associated with antibiotic usage [75] .

### Infant antibiotic administration and childhood hay fever

#### Study characteristics

The 9 studies (supplementary tables S3c and S3d) examining hay fever as outcome were conducted in Sweden (2), China (1), Turkey (1), Japan (1), Taiwan (1), United Stated (1), United Kingdom (1) and Colombia (1). Six were cohort and three were cross-sectional studies. The sample sizes ranged from 1550- 13,335 children. The children’s age of hay fever outcome ranged from 6+ months of age till 8 years.

The prevalence of hay fever varied from 8.7% at 7.5 years of age to 42.7% at 4-6 years of age. Hay fever that had been diagnosed by a physician tended to have lower prevalences than hay fever that was self-reported (doctor-diagnosed: 8.1% versus self-reported: 29.2% (Tamay, 2014); doctor-diagnosed: 12.6% versus self-reported: 42.7% (Wang, 2016)).

#### Main findings

Six of the nine publications reported a significant relationship between early antibiotics and childhood hay fever. Significant OR effect sizes ranged from OR 1.23 (1.09-1.40) to OR 1.75 (1.03-2.97) and significant HR effect sizes ranged from HR 1.41 (1.35-1.47) to HR 1.75 (1.72-1.78). One publication containing two sub-studies, found that there was no significant relationship between antibiotics and hay fever at the age of 4 (OR 1.0 (0.9-1.3)), nor at the age of 8 (OR 1.0 (0.8– 1.2)) [68].

Two more publications reported both insignificant and significant relationships: one study found a significant relationship at 6-7 years of age, but not at 13-14 years of age [81] ; another publication with two sub-studies of children born in 1998 and 2003 respectively, only found a significant relationship in 1998 [66].

#### Influential factors

##### Confounding by indication and by other factors

Potential confounders generally taken into account were family history of allergies, gender, smoking, pets and number of siblings. Important potential confounders not always taken into account were delivery mode (examined in 5/9 studies), infectious diseases (examined in 5/9 studies) and prenatal antibiotics (never taken into account). Five publications took confounding by indication at least somewhat into account, of which three of these reported significant relationships and one reported a significant effect in one of the two cohorts they had studied.

The majority of publications did not examine the presence of a dose-response relationship. Of the studies that did, two found a dose-response relationship and one study found no dose-response relationship. Hoskin-Parr et al.,(2013) [67] found a significant relationship with hay fever only in children who had had at least 4 courses of antibiotics. Yamamoto-Hanada et al. (2017) [65] found that the significant relationship they found between antibiotics and hay fever was driven only by cephalosporins. Wang et al., (2013) [66] stratified their study population according to having had respiratory tract infections or not, and found the relationship between antibiotics and hay fever to be significant only in the sample of children without respiratory tract infections.

### Quality assessments of publications on prenatal and infant antibiotic exposure and childhood allergies

Using the risk of bias tool, the Newcastle Ottawa Scale (NOS), the majority of studies obtained a relatively high score (usually scoring a 6 or 7 out of 9), indicating generally well-conducted studies with a medium level of risk (supplementary tables S2a, 2b, Supplementary Tables S3a, 3b and 3c). Points were mainly lost due to inadequate correcting for relevant potential confounders (such as maternal infections, delivery mode, genetic factors, postnatal antibiotics and childhood infections), outcomes being self-reported instead of physician-diagnosed, not addressing missing data or those lost to follow-up, or small sample sizes.

## Discussion

The aim of this systematic review was to collect and assess the available evidence accumulated over the last 10 years regarding the relationship between prenatal and infant antibiotic exposure and the onset of the childhood allergies, asthma, eczema and hay fever from the ages of 0-18 years of age.

### Childhood asthma

#### Prenatal antibiotic exposure

The majority of studies on prenatal antibiotics reported significant relationships between prenatal antibiotics and childhood asthma. Most authors concluded that antibiotics were likely to play a causal role, while the authors of three studies did not believe that the significant associations they had found were due to antibiotics themselves (Loewen, 2018; Stokholm, 2014 and Ortqvist, 2014). Loewen et al. (2018) and Stokholm et al.,(2014), whose studies were assessed to be of low risk based on the NOS (8 out of 9 points), observed similar significant relationships when examining maternal antibiotic usage in the periods before and after pregnancy, as well as during pregnancy. These findings led the authors to conclude that the actual relationship with the child’s asthma may have to do with the mothers’ general susceptibility for infections, which she may have transferred to her child. It is also possible as Blaser et al., (2014) [83] had suggested in response that pre-pregnancy antibiotic usage led to an altered maternal microbiome before pregnancy, and this new composition of bacteria transferred to the child during delivery. Blaser et al. also commented that the effects of maternal postnatal antibiotic usage may have been passed on to the child through breastfeeding. As these were the only two studies examining maternal antibiotic usage prior to, during, and after pregnancy, their findings that the association was not specific to pregnancy call for more research. The third study concluding that antibiotics were unlikely to be causal, had conducted both a population study and an additional sibling-case study (Ortqvist, 2014). In this sibling study, assessed to be of medium risk based on the NOS (7 out of 9), the relationship between prenatal antibiotic exposure and asthma was not significant, leading the authors to conclude that previously found associations were confounded by familial genetic and environmental factors. Many genes have been identified making individuals susceptible to asthma, and twin studies have already shown that asthma has a substantial genetic basis [84, 85].

#### Infant antibiotic administration

Over half of the studies (17/27) examining early life antibiotics and childhood asthma reported a significant association and ten studies reported either inconsistent findings or lower to insignificant effect sizes after taking reverse causation or confounding by indication into account. Earlier systematic reviews and meta-analyses also concluded that antibiotic exposure was somewhat associated with asthma, but that reverse causation and confounding by respiratory diseases explained at least part of the associations observed in many studies [24, 25, 27].

The proportion of studies reporting significant associations between antibiotic exposure and asthma was higher with prenatal than with infant antibiotic exposure. However, the four studies that had examined both prenatal as well as infant antibiotic exposure and asthma, found higher effect sizes for the latter (Yoshida, 2018; Metsala, 2015 ; Ortqvist, 2014; Martel, 2009). The range of significant effect sizes in both prenatal and early life antibiotics were similar (i.e. (prenatal) OR 1.08 to OR 3.19 versus (infant antibiotic administration) OR 1.12 to OR 3.21). It is therefore unclear what type of exposure may have the greatest impact on the development of asthma.

Studies investigating asthma onset at different ages found that the effect of antibiotics was always stronger in younger age groups than older age groups, suggesting a higher risk of reverse causation, where the first symptoms of asthma may have been treated by antibiotics [25]. Alternatively, antibiotics may exert the greatest effect on the microbiome shortly after exposure, inducing asthma symptoms at earlier ages [43].

### Childhood eczema

#### Prenatal and infant antibiotic exposure

Studies investigating the relationship between prenatal antibiotics and eczema were scarce. One publication reported a significant relationship in their main findings, one reported a significant relationship when the intra-partum antibiotic exposure lasted more than 24 h and the other reported a significant relationship only when the mother was atopic and had taken antibiotics throughout pregnancy.

About half of the studies on infant antibiotic administration in this review showed significant relationships, half showed insignificant relationships (one study found an almost protective effect of antibiotics for eczema) and a few studies had inconclusive findings. Of the eight studies that had taken confounding by indication or reverse causation at least somewhat into account, just two showed significant relationships. This indicates there may still be too little evidence to conclude that early antibiotic usage increases the risk of childhood eczema. An earlier meta-analysis, published in 2013 [26] showed an insignificant pooled relationship between prenatal antibiotics and eczema, based on three studies (OR 1.30 (0.86-1.95)) and a significant pooled relationship between postnatal antibiotics and eczema, based on 17 studies (OR1.41 (1.30-1.53). Many of these studies, however, did not take reverse causation and confounding by indication into account. Additionally, no mention was made in the review about whether intra-partum antibiotics had been included as part of the antibiotics exposure.

### Childhood hay fever

#### Prenatal and infant antibiotic exposure

The number of studies examining antibiotics and hay fever was relatively scarce (none for prenatal antibiotic exposure and ten for infant antibiotic administration), but these few findings provided some evidence for a relationship between infant antibiotic administration and childhood hay fever. The authors of just one of the nine publications concluded there was no significant relationship, although several others had mixed findings and reported significant relationships under certain conditions. A recent meta-analysis of publications identified till November 2015 on infant antibiotic administration, found significant pooled odds ratios of 1.25 (1.03-1.52) for eczema and 1.23 ( 1.08-1.41) for hay fever, after selecting studies that had taken reverse causation into account (8/22 for eczema and 6/22 for hay fever) [28]. More than half of the studies investigated in that review individually reported insignificant results, but the pooling of findings still resulted in a significant effect. However, the individual studies varied greatly in the number and types of confounders taken into consideration.

Although confounding by indication through respiratory diseases are more obvious and more frequently considered with the outcome asthma, children with eczema and hay fever are also more likely to have skin, gastro-intestinal, ear-nose-throat, respiratory and other infectious diseases [86], which in turn are often treated with antibiotics [23] It is therefore important when investigating eczema and hay fever to take confounding by indication by infectious diseases into account as well.

### Microbiota hypothesis

The significant findings reported in these publications between prenatal antibiotic exposure or infant antibiotic administration and childhood allergies fall in line with the microbiota hypothesis which posits that disruptions to the microbial composition during a critical period in early life, can have long-lasting effects on the immune system [87]. A newborn’s immune system leans towards a Th2 phenotype which allows microbial colonization and helps to avoid inflammatory responses to harmless microbes. Under normal circumstances, there is a gradual shift from the Th2 to Th1 phenotype, when the immune system encounters pathogens and then elicits inflammatory responses. The early immune system is therefore ‘educated’ about when to tolerate microbes and when to elicit inflammatory responses. Disturbances to the microbiome can cause delay or disorder to this phenotype shift and promote a strong immune response to harmless microbes, leading to tissue damage and disruption of the normal development of the immune system [88]. Antibiotic treatment (vancomycin) to neonatal mice caused shifts in gut microbiota and made them susceptible to indicators of allergic asthma, whereas no significant effect occurred when administered to adult mice [89]. This supports a critical window in early life when disruption of the microbiome can have long-lasting effects.

This disruption of the microbiome can start during pregnancy, when the development of the child’s immune system is already underway. The first bacteria to colonize human beings are most likely transmitted prenatally from the maternal gut through the placenta and the amniotic fluid [19, 90]. Stokholm et al. (2014) [91] analysed vaginal microbiome samples at 36 weeks of pregnancy and found that women who had received any antibiotics during pregnancy had an increased colonization of Staphylococcus species compared to women who had not had any antibiotics during pregnancy. Increased vaginal Staphylococci is in turn associated with asthma in later childhood [92].

Similarly, disruptions to the microbiome after birth in early infancy caused by antibiotics can impede the normal development of the immune system. Allergic children tend to have different bacterial compositions in the gut than non-allergic children, such as fewer Bacteroides and Bifidobacteria, and more *Staphylococcus aureus* and *Clostridium difficile* [93]. Arrieta et al., (2015) [94] found that the bacterial genera Lachnospira, Veillonella, Faecalibacterium, and Rothia were significantly decreased in the first 100 days after birth in children who later went on to develop asthma. Low abundance of Bacteroidetes and greater abundance of Clostridia at 18 months was associated with later eczema [95]. Bisgaard et al., (2011) [96] found a reduced diversity and overgrowth with Staphylococci at one month in school aged children who later developed hay fever.

This review found that broad-spectrum antibiotics, such as macrolides, had the strongest effects possibly due to causing the greatest disorder to the microbiome. An experimental study showed that early life administration of a single macrolide course in mice led to long-lasting modifications of the gut microbiota and immune system [97].

### Quality assessment of studies

Although the majority of studies scored relatively well according to the NOS, this score did not reflect the consequences of confounding by indication. Most studies lost one out of two possible points in the ‘comparability’ category, which could indicate confounding by indication. For studies in this review that are prone to confounding by indication, the ‘comparability’ category (in which a maximum of 2 points was possible) may weigh more in the risk of bias measurement than the other categories. The ‘selection’ category was relatively easy to gain points for, subsequently compensating for a lack of points gained in the other two categories. Other issues we believed could not be captured by the NOS were missing information about intra-partum antibiotics in the exposure and lack of clarity at times about how analyses had been conducted and which covariates were included. The NOS quality scores may be somewhat higher than we may have judged in general, due to the individual scoring components not being of equal value for this particular review and not being able to deduce points for other important issues.

The heterogeneity of all the studies added to the complexity of making comparisons across studies, assessing the actual associations between early life antibiotics and the various allergies, and determining whether the associations are indeed causal. Antibiotics exposure was measured in different ways (self-reported or from medical databases) and at various ages (for example during the first 6 months or during the first year). There were different follow-up periods; some studies measured the outcome directly following the exposure period, and other studies allowed many years between exposure and outcome. Allergy outcomes also differed in how they were measured (self-reported with varying definitions, physician-diagnosed, or identified in a medication database). Allergies were measured at different childhood ages and spanned different periods (eg . ‘current asthma’ (last 12 months), or ‘ever asthma’ (up to 5 years of age)). The number and types of potential confounding factors also differed substantially per study, making the comparability of findings more challenging.

### Strengths and limitations

Strengths of this review include providing a complete overview of all current evidence related to the relationship of early life antibiotic exposure and three common allergies. Our detailed examination of influential factors helps to increase the understanding of this complex relationship, in particular the possible influential factors that need to be taken into account in further studies. Although scoring using the NOS, revealed most studies to be of a medium level of risk, important limitations including the heterogeneity of studies and missing information on intra-partum antibiotics, made the comparison of studies unsuitable for a meta-analysis or definite conclusions. We searched only two main databases, Pubmed and Embase, leaving the possibility of having missed publications. However, we believe it is highly likely that these two large databases captured all the relevant literature on these topics: our search through the reference lists for additional titles will also have decreased the chance of missing important papers. Other limitations are the exclusion of grey literature and inclusion of only English publications for review. Including unpublished data may mitigate publication bias, but can introduce other types of bias. We chose to improve the chances of higher quality and comparable studies by only including peer-reviewed publications. Although we believe we included all relevant publications, there is still a chance of having missed a publication written in another language.

### Implications

Many risk factors are associated with asthma and other allergies, meaning that multiple interventions may need to be put into place to address these chronic diseases. Abreo et al. (2018) [10] calculated the population attributable fractions (PAFs) of a large range of potentially modifiable risk factors for childhood asthma and found 51% of asthma cases to be attributable to RSV lower respiratory tract infection and antibiotic use. The increasing numbers of bacteria which are becoming resistant to antibiotics [98] and the possible risks of a range of chronic diseases associated not only with allergies, but with overweight/obesity, autoimmune diseases and neurological disorders (12–14), provide enough reason to be more conservative with regard to prescribing antibiotics for pregnant women and children, or at the very least prescribe narrow- instead of broad-spectrum antibiotics. A recent study comparing children treated for acute respiratory tract infections with narrow- or broad-spectrum antibiotics, found no differences in recovery between the two types of antibiotics, and that broad-spectrum antibiotics resulted in higher adverse events [99].

Another implication may be to explore the possibilities of probiotic supplementation to counteract the effects of disturbances to the microbiome. There has been quite some evidence for probiotic administration with respect to reducing the effects of antibiotics leading to antibiotic-associated diarrhea (AAD) [100] and for reducing the risk of atopic dermatitis [101] and hay fever [102]. However, as of yet, meta-analyses have not found probiotics to be preventive of asthma [103, 104].

Further implications involve the development of well-designed studies, taking issues such as intra-partum antibiotics, maternal and childhood infections and familial allergies into account. The most frequent cause of antibiotic exposure during pregnancy is due to intra-partum antimicrobial prophylaxis (IAP); it is estimated that over 30% of all pregnant women receive intra-partum antibiotics during pregnancy There is evidence that intra-partum antibiotics can cross the placenta to enter the infant’s bloodstream and stay there for several hours after administration [105]. Azad et al, (2016 ) [19], for example, found that infants who had been exposed to intra-partum antibiotics for either prevention of GBS or CS related infections, had fewer bacteroides species and higher numbers of Clostridium and Enterococcus at 3 months of age.

Some studies found the strongest effects were found by antibiotics used for respiratory tract infections compared to urinary tract infections (Stokholm, 2014; Ortqvist, 2014; Metsala, 2014), lending some weight to the argument that there may be some confounding by maternal respiratory infections. As broad-spectrum antibiotics are given more often for respiratory infections than urinary tract infections, another explanation is that their greater impact on the microbiome could increase the chance of allergies. It is important for future studies to examine the independent effects of maternal infections during pregnancy on the development of childhood asthma; a meta-analysis by Zhu et al. (2016) [106] found that prenatal maternal infections (particularly fever episodes and urogenital infections) were associated with childhood asthma and eczema, some of these studies had been corrected for prenatal antibiotic usage.

## Conclusions

The data collected for this review point to a moderate amount of evidence for a relationship between early life antibiotics (in particular prenatal antibiotic exposure) and childhood asthma, some evidence for a relationship with hay fever and less convincing evidence for a relationship with eczema. The high proportion of studies finding significant relationships, as well as dose-response relationships between prenatal or childhood antibiotics and childhood asthma, corroborated by experimental animal and human microbial studies, support a causal role of antibiotics in the development of childhood asthma. More studies are still needed addressing intra-partum antibiotics, familial factors, and possible confounding by maternal and childhood infections to be able to draw conclusions with greater confidence. Children with multiple, broad-spectrum antibiotic exposures early in life (prenatal and during early infancy) appear to have a greater risk of allergies, especially asthma; these effects should be investigated further.

## Supplementary information

**Additional file 1.** Supplementary data_data extraction tables: This file contains tables of the data collected from the publications examining (1) the relationship between prenatal antibiotic exposure and the childhood allergies asthma and eczema (Tables S2a and S2b) and (2) the relationship between infant antibiotic administration and the childhood allergies asthma, eczema and hay fever (Tables S3a, S3b, S3c and S3d).

**Additional file 2.** Supplementary data_search strategy and process: This file has a table containing the search terms and strategy used for Embase and Pubmed to retrieve relevant publications (Table S1) and a flowchart depicting the search process (Figure S1).

## Data Availability

Not applicable, as no original datasets were generated or analysed during the current study.

## Abbreviations

OR: Odds ratio
HR: Hazard ratio
RR: Relative risk
PAF: Population attributable fraction
Th1/Th2: T helper cell type 1/ T helper cell type 2
RSV: Respiratory syncytial virus
CS: Caesarean section
GBS: Group B Streptococcus
IAP: Intra-partum antimicrobial prophylaxis
AAD: Antibiotic-associated diarrhea
NOS: Newcastle-Ottawa Scale
