# Supplementary material for: The relationship of prenatal antibiotic exposure and infant antibiotic administration with childhood allergies: a systematic review
Source: BMC Pediatr. 2020 Jun 27;20:312. doi: 10.1186/s12887-020-02042-8 (PMC7320596; doi:10.1186/s12887-020-02042-8)
Supplement: Supplementary file 1 — Additional file 1. Supplementary data_data extraction tables: This file contains tables of the data collected from the publications examining (1) the relationship between prenatal antibiotic exposure and the childhood allergies asthma and eczema (Tables S2a and S2b) and (2) the relationship between infant antibiotic administration and the childhood allergies asthma, eczema and hay fever (Tables S3a, S3b, S3c and S3d). [file 12887_2020_2042_MOESM1_ESM.docx]

**Table S2a**. Prenatal antibiotic exposure and childhood asthma: study characteristics and findings

S: study CS: caesarean section RC: reverse causation OR: odds ratio HR: hazard ratio RR: relative risk IR: incidence rate NOS: Newcastle-Ottawa Scale (8-9 low risk; 6-7 medium risk; <6 unclear)

| **Study/ authors’ conclusions (relevant for this review)** | **Country** | **Population** | **-Timing antibiotic**  **exposure**  **-Prevalence of**  **exposure** | **Health outcome, age and prevalence** | **Summary measures (HR, RR or OR)** | **Influential factors** | | **Risk of bias**  **Using the**  **NOS**^31^  8-9: low risk  6-7: medium risk  <6: unclear risk |
| --- | --- | --- | --- | --- | --- | --- | --- | --- |
| ***Asthma*** | | | | | | | |  |
| **Loewen, 2018**^34^  Retrospective cohort study  *Dose response relationship between prenatal antibiotics and asthma, pre-pregnancy and postnatal antibiotics also associated with childhood asthma. Association is not specific to pregnancy.* | Canada:  All children born in Manitoba | 213,661 mother-child dyads  Study period: 1996-2012 | - Whole pregnancy till date of birth + period of nine months before pregnancy and period of nine months after pregnancy  -Prevalence during pregnancy: 36.8%  -Prevalence 9 months before pregnancy: 35.2%  -Prevalence 9 months after pregnancy: 35.5% | -Asthma  -5+ years (median 9.5)  -Prevalence: 10.1% | HR:1.23 (1.20-1.27)  Maternal antibiotics before and after pregnancy also significantly associated with asthma | *Dose-response*  *relationship* | Yes | Selection: 4/4  Comparability:1/2  Outcome:3/3  Total: 8 (low) |
|  |  |  |  |  |  | *Intra-partum*  *Antibiotics* | No |  |
|  |  |  |  |  |  | *Types of antibiotics* | Beta-lactam penicillins, macrolides, lincosamides, and streptogramins, sulphonamides and trimethoprim and (weakly) tetracyclines, aminoglycosides and quinolones associated with asthma |  |
|  |  |  |  |  |  | *Childhood antibiotics usage* | Adjusted for |  |
|  |  |  |  |  |  | *Gender* | IR males higher effect size, adjusted for |  |
|  |  |  |  |  |  | *Birth weight* | IR <3000 gr higher effet size than 3000+ gr. |  |
|  |  |  |  |  |  | *Delivery mode* | IR CS higher, not adjusted for |  |
|  |  |  |  |  |  | *Family allergies* | IR maternal asthma higher effect size, adjusted for |  |
|  |  |  |  |  |  | *Breastfeeding* | IR lower, not adjusted for |  |
|  |  |  |  |  |  | *Maternal infections* | Not examined |  |
|  |  |  |  |  |  | *Childhood infections* | Not examined |  |
| **Yoshida, 2018**^35^  Two cohorts:  S1: population cohort  S2: Matched sibling cohort study  *Authors describe relationship between prenatal exposure and asthma as being weak, particularly in comparison to exposure in 1st year of life, but the relationship is still significant.* | Japan: | S1: 83470 children  S2: 20630  Study period: 2005-2014 | -Period starting from 9 months before birth month, unclear if intra-partum antibiotics are included  -S1:Prevalence: 24.7%  -S2: Prevalence: 26.3% | -Asthma  -1-6 years old  -S1: Prevalence: 4.4%  -S2: Prevalence: 5.6% | -S1:  1-<3 years:  HR:1.18 (1.08-1.30)  3-<6 years:  HR:1.09 (0.97- 1.22)  -S2: 1-<6 years: HR:1.34  (1.05-1.72)  Timing of outcome: C1 showed significant and stronger effect at an earlier age. | *Dose-response*  *relationship* | S1: yes ; S2: sign. in 1-2 prescriptions (not more) | Selection: 4/4  Comparability:1/2  Outcome:2/3  Total:7 (medium) |
|  |  |  |  |  |  | *Intra-partum*  *antibiotics* | Unclear |  |
|  |  |  |  |  |  | *Types of antibiotics* | S1: only cephalosporin sign. in 1-3 year olds  CS: no differences between types |  |
|  |  |  |  |  |  | *Child postnatal*  *antibiotic usage* | Not examined |  |
|  |  |  |  |  |  | *Gender* | Males: effect sign. higher |  |
|  |  |  |  |  |  | *Birth weight* | Not examined |  |
|  |  |  |  |  |  | *Delivery mode* | Not examined |  |
|  |  |  |  |  |  | *Family allergies* | Matched sibling cohort adjusts for family genetic and other shared factors |  |
|  |  |  |  |  |  | *Breastfeeding* | Not examined |  |
|  |  |  |  |  |  | *Maternal infections* | Not examined |  |
|  |  |  |  |  |  | *Childhood infections* | Not examined |  |
| **Kashanian**, **2017**^36^  Case-control study  *Prenatal antibiotic exposure one of several variables predictive of asthma besides maternal asthma, vaginal bleeding, older maternal age >30 and breastfeeding (protective), with maternal asthma having the greatest effect.* | Iran | 134 kids with asthma, 134 kids without asthma  Study period: 2009-2014 | -Pregnancy and delivery  -Kids with asthma  Prevalence exposure: 29.9%  -Kids without asthma: prevalence exposure: 9.0% | -Asthma  7-14 years old | OR: 3.19 (1.52-6.67) | *Dose-response relationship* | Not examined | Selection: 4/4  Comparability:1/2  Exposure:1/3  Total: 6 (medium) |
|  |  |  |  |  |  | *Intra-partum*  *Antibiotics* | Unclear |  |
|  |  |  |  |  |  | *Types of antibiotics* | Not examined |  |
|  |  |  |  |  |  | *Child postnatal*  *antibiotic usage* | Not examined |  |
|  |  |  |  |  |  | *Gender* | Not examined |  |
|  |  |  |  |  |  | *Birth weight* | Examined univariably, not significant |  |
|  |  |  |  |  |  | *Delivery mode* | Examined univariably, not significant |  |
|  |  |  |  |  |  | *Family allergies* | Maternal asthma independently predictive |  |
|  |  |  |  |  |  | *Breastfeeding* | Predictive (protective) |  |
|  |  |  |  |  |  | *Maternal infections* | Not examined |  |
|  |  |  |  |  |  | *Childhood infections* | Not examined |  |
| **Mulder, 2016^37^**  Two cohorts:  S1:case-sibling  S2:case-control  *Third trimester exposure associated with a small increased risk of childhood asthma. Time invariant confounders or time trends in antibiotic exposure did not affect this relationship.* | Netherlands | S1:Cases: 3754  Control: 22523  S2: 1228 kids with asthma , 1228 siblings without asthma  Data from prescription database, but no mention of any study period. | -Pregnancy period: birthdate minus 273 days  S1: Cases: prevalence exposure: 25.4%  Controls: prevalence exposure: 17.8%  S2: Cases: prevalence exposure: 24.5%  Siblings: prevalence exposure: 22.3% | -Asthma  0-5 years | S1: Case-control:  OR:1.45 (1.33-1.58)  S2: Case-sibling:  OR: 1.06 (0.85–1.32)  Third trimester antibiotics only:  S1: Case-control: OR:1.40 (1.25–1.57)  S2: Case-sibling: OR 1.37 (1.02-1.83) | *Dose-response*  *relationship* | Not examined | Selection: 4/4  Comparability:1/2  Exposure:3/3  Total: 8 (low) |
|  |  |  |  |  |  | *Intra-partum*  *Antibiotics* | Unclear |  |
|  |  |  |  |  |  | *Types of*  *antibiotics* | S1: Beta-lactam penicillins, sulphonamides and trimethoprim significant.  S2: no differences in antibiotic types |  |
|  |  |  |  |  |  | *Child postnatal antibiotic usage* | Not examined (to not underestimate effects) |  |
|  |  |  |  |  |  | *Gender* | Adjusted for |  |
|  |  |  |  |  |  | *Birth weight* | Not examined |  |
|  |  |  |  |  |  | *Delivery mode* | Not examined |  |
|  |  |  |  |  |  | *Family allergies* | Sibling-control study adjusted or family genetics and other shared factors |  |
|  |  |  |  |  |  | *Breastfeeding* | Not examined |  |
|  |  |  |  |  |  | *Maternal infections* | Not examined |  |
|  |  |  |  |  |  | *Childhood infections* | Not examined |  |
| **Wu, 2016**^38^  Population birth cohort study  *Maternal UTI infections and antibiotic exposure are one of various predictors associated with asthma, the strongest being infant antibiotic use, infant health care visits, not having older siblings* *and maternal asthma. The effects are cumulative (i.e. maternal UTIS, CS section and multiple postnatal antibiotics resulted in stronger effects on asthma)* | United States | 136098 children  Children born between 1995 and 2003 | -Maternal urinary tract infection throughout pregnancy, prevalence UTI: 13.8%  -Subgroup: maternal antibiotic usage | -Asthma  -6 years old  -Prevalence: 13.3% | -Urinary tract infections (UTI). OR: 1.34 (1.28-1.40).  Per additional UTI. OR: 1.04 (1.02-1.07)    -Antibiotics. OR: 1.13 (1.12-1.15). Per additional course OR: 1.06 (1.05-1.08) | *Dose-response*  *relationship* | Yes | Selection: 4/4  Comparability:1/2  Outcome:2/3  Total:7 (medium) |
|  |  |  |  |  |  | *Intra-partum*  *Antibiotics* | Unclear |  |
|  |  |  |  |  |  | *Types of*  *antibiotics* | Not examined |  |
|  |  |  |  |  |  | *Child postnatal*  *antibiotic usage* | Infant antibiotics use is a predictor (besides prenatal antibiotic exposure) |  |
|  |  |  |  |  |  | *Gender* | Adjusted for |  |
|  |  |  |  |  |  | *Birth weight* | Adjusted for |  |
|  |  |  |  |  |  | *Delivery mode* | CS is a significant predictor |  |
|  |  |  |  |  |  | *Family allergies* | Maternal asthma is a predictor |  |
|  |  |  |  |  |  | *Breastfeeding* | Not examined |  |
|  |  |  |  |  |  | *Maternal infections* | Maternal urinary tract infections is an independent predictor |  |
|  |  |  |  |  |  | *Childhood infections* | Severe bronchiolitis health care encounters and chronic lung disease not predictive |  |
| **Chu, 2015**^39^  Prospective birth cohort study  *Maternal exposure to penicillins or chloramphenicols during pregnancy is associated with childhood asthma by 7 years of age.* | United States | 39,907  Study period:  1959-1965 | 28 days before last menstrual period (LMP) till birth. Antibiotic use reported by mothers during prenatal visits, therefore intra-partum antibiotics usage may not be included (although other information is included , such as delivery mode)  Prevalence: 26.4% | -Asthma  -7 years old  -Prevalence: 5.5% | OR: 1.13 (1.02-1.24) | *Dose-response*  *relationship* | Not examined | Selection: 4/4  Comparability:1/2  Outcome:3/3  Total:8 (low) |
|  |  |  |  |  |  | *Intra-partum*  *Antibiotics* | Unclear |  |
|  |  |  |  |  |  | *Types of*  *antibiotics* | Only penicillins and chloramphenicols significant. |  |
|  |  |  |  |  |  | *Child postnatal*  *antibiotic usage* | Not examined |  |
|  |  |  |  |  |  | *Gender* | Univariably examined, significantly more males than females in asthma sample |  |
|  |  |  |  |  |  | *Birth weight* | Univariably examined, somewhat higher proportion of birth weight <2500 grams |  |
|  |  |  |  |  |  | *Delivery mode* | No differences when examined univarriably |  |
|  |  |  |  |  |  | *Family allergies* | Maternal asthma significantly more likely in asthmatic children, included as confounder |  |
|  |  |  |  |  |  | *Breastfeeding* | Not examined |  |
|  |  |  |  |  |  | *Maternal infections* | Not examined |  |
|  |  |  |  |  |  | *Childhood infections* | Not examined |  |
| **Lapin, 2015**^40^  Prospective cohort study  *This study suggests an association between prenatal antibiotic*  *use and the development of asthma in at-risk children by 3 years of age. (There was no relationship with wheezing.)* | United States | 298 children from families at risk of asthma in disadvantaged area  Recruited from 1998 to 2004 | -Antibiotic use at various moments throughout pregnancy (intra-partum antibiotics not included)  -Prevalence: 35% | -Asthma  -3 years old  -Prevalence: 14.8% | OR: 3.12(1.44-6.77) | *Dose-response relationship* | Not examined | Selection: 3/4  Comparability:1/2  Outcome:1/3  Total:5 (unclear) |
|  |  |  |  |  |  | *Intra-partum*  *Antibiotics* | No |  |
|  |  |  |  |  |  | *Types of antibiotics* | Not examined |  |
|  |  |  |  |  |  | *Child postnatal*  *antibiotic usage* | Postnatal antibiotic use for respiratory infections examined in children, is an independent predictor. Subgroup of children who did not take antibiotics for respiratory infections, showed sign. relationship between antibiotics and asthma. |  |
|  |  |  |  |  |  | *Gender* | Univariably examined, no significant difference |  |
|  |  |  |  |  |  | *Birth weight* | Univariably examined, no significant difference |  |
|  |  |  |  |  |  | *Delivery mode* | Not examined |  |
|  |  |  |  |  |  | *Family allergies* | Maternal asthma independent predictor. Subgroup of mothers without asthma showed strong significant effect (OR 5.75) of prenatal antibiotics and asthma |  |
|  |  |  |  |  |  | *Breastfeeding* | Univariably examined, no significant difference |  |
|  |  |  |  |  |  | *Maternal infections* | Not examined |  |
|  |  |  |  |  |  | *Childhood infections* | Infections not examined but antibiotics taken by children for respiratory diseases, see childhood antibiotics |  |
| **Stokholm, 2014**^41^  Prospective registry-based cohort study  *Maternal antibiotic use may be a surrogate marker of a mother’s general propensity for infections as the underlying link*  *between a mother’s use of antibiotics and risk of asthma in the offspring* | Denmark | 910301 (all Danish children)  Children born between  1997 and 2010 | -Period throughout whole pregnancy  (80 weeks before and 80 weeks after pregnancy also examined).  -Prevalence: 32% | Three types of outcomes measuring asthma  -All ages in childhood  -Ages above 6 | All childhood ages:  Inpatient admission: IRR 1.24 (1.18-1.30)  Outpatient attendance: IRR 1.22 (1.18-1.26)  Inhaled corticosteroid use:  IRR 1.18 (1.15-1.20)  Ages 6+:  Inpatient admission: IRR 1·29 (1·08–1·55)  Outpatient attendance: IRR 1·09 (1·02–1·17)  Inhaled corticosteroid use:  1·06 (1·02– 1·11)  80 pre- pregnancy and 80 weeks post-pregnancy also showed similar significant relationships | *Dose-response*  *relationship* | Yes, not only in pregnancy, but in period from 80 weeks before pregnancy and period till 80 weeks after pregnancy | Selection: 4/4  Comparability:1/2  Outcome:3/3  Total:8 (low) |
|  |  |  |  |  |  | *Intra-partum*  *Antibiotics* | Unclear |  |
|  |  |  |  |  |  | *Types of antibiotics* | All antibiotics significant, but antibiotics for respiratory infections had strongest effects |  |
|  |  |  |  |  |  | *Child postnatal*  *antibiotic usage* | Not examined |  |
|  |  |  |  |  |  | *Gender* | Adjusted for |  |
|  |  |  |  |  |  | *Birth weight* | Adjusted for |  |
|  |  |  |  |  |  | *Delivery mode* | Adjusted for |  |
|  |  |  |  |  |  | *Family allergies* | Adjusted for maternal asthma. Stratifications according to maternal asthma showed similar significant relationships. |  |
|  |  |  |  |  |  | *Breastfeeding* | Not examined |  |
|  |  |  |  |  |  | *Maternal infections* | Antibiotics used to treat respiratory diseases examined, not infections themselves. |  |
|  |  |  |  |  |  | *Childhood infections* | Not examined |  |
| **Metsala, 2014**^42^  Population and register-based case-control study  *Prenatal and post-natal exposure to antibiotics was associated with an increased risk of asthma among Finnish children* | Finland | 6690 case-control pairs  Children born between 1996 and 2004 | -Antibiotic purchases throughout pregnancy including the date of birth.  -Control mothers: prevalence 24% | -Diagnosis 3-9 years  -Diagnosis: 3-5 years  -Diagnosis: 6-9 years | -Any OR:1.31 (1.21-1.42)  -3-5 years OR: 1.32 (1.21–1.46)  -6-9 years OR1.23 (1.04–1.48)  Timing age of onset: higher effect size at younger age of onset | *Dose-response*  *relationship* | Yes | Selection: 4/4  Comparability:1/2  Exposure:2/3  Total:7 (medium) |
|  |  |  |  |  |  | *Intra-partum*  *Antibiotics* | Unclear |  |
|  |  |  |  |  |  | *Types of antibiotics* | Prenatal cephalosporins, macrolides, penicillins with extended spectrum and phenoxymethylpenicillin  associated with an increased risk of asthma. |  |
|  |  |  |  |  |  | *Child postnatal*  *antibiotic usage* | Not adjusted for in prenatal antibiotics model (but prenatal antibiotics were adjusted for in post-natal antibiotics model) |  |
|  |  |  |  |  |  | *Gender* | Not examined |  |
|  |  |  |  |  |  | *Birth weight* | Not examined |  |
|  |  |  |  |  |  | *Delivery mode* | Not associated with both antibiotics and asthma, therefore not adjusted for |  |
|  |  |  |  |  |  | *Family allergies* | Adjusted for maternal asthma |  |
|  |  |  |  |  |  | *Breastfeeding* | Not examined |  |
|  |  |  |  |  |  | *Maternal infections* | Infections themselves not examined but antibiotics for gram-positive bacteria were significant, for urinary tract infections not significant, for both, the strongest effect. |  |
|  |  |  |  |  |  | *Childhood infections* | Not examined |  |
| **Ortqvist, 2014**^43^  S1: Prospective cohort study  S2: Sibling-control analysis  *Relationship between prenatal antibiotic exposure and asthma is mainly explained by confounding through familial factors* | Sweden | S1: 493,785 full cohort  S2: 180,894 sibling analysis  Children born between 2006 and 2010 | -Prenatal exposure 1^st^, 2^nd^, 3^rd^ trimester till birth.  (register does not contain prescriptions obtained in hospitals, so unclear if this included intra-partum antibiotics)  Prevalence cohort: 20%  Prevalence sibling cohort: 16%  Timing: neither sub-study showed any differences in effect according to timing of exposure during pregnancy | -Asthma  -All ages till about 5 years  Prevalence outcome cohort: 6% | S1: Cohort analysis:  HR: 1.28 (1.25-1.32)  S2: Sibling analysis:  HR: 0.99 (0.92-1.07) | *Dose-response*  *relationship* | Not examined | Selection: 4/4  Comparability:1/2  Outcome:2/3  Total:7 (medium) |
|  |  |  |  |  |  | *Intra-partum*  *Antibiotics* | Unclear |  |
|  |  |  |  |  |  | *Types of antibiotics* | S1: Antibiotics for respiratory tract infections somewhat greater effect size than for urinary tract infections (both significant)  S2: Neither type of antibiotic was significant |  |
|  |  |  |  |  |  | *Child postnatal*  *antibiotic usage* | Not examined |  |
|  |  |  |  |  |  | *Gender* | Not examined |  |
|  |  |  |  |  |  | *Birth weight* | Not examined |  |
|  |  |  |  |  |  | *Delivery mode* | Not adjusted for in prenatal model (only postnatal model) |  |
|  |  |  |  |  |  | *Family allergies* | S1: Adjusted for maternal asthma  S2: Sibling analysis unlike cohort study showed an insignificant relationship. |  |
|  |  |  |  |  |  | *Breastfeeding* | Not examined directly, antibiotics for certain indications were examined, see types of antibiotics |  |
|  |  |  |  |  |  | *Maternal infections* | Not examined |  |
| **Stensballe, 2013**^44^  Two cohorts, but focus on 2^nd^ cohort for this SR.  National birth cohort  *Prenatal antibiotics are associated with increased risk of childhood asthma, also in samples of*  *antibiotic use for non-respiratory infections, suggesting no confounding by mother’s asthma status. No significant association*  *with eczema.* | Denmark | - 30675 children  Children born between 1997 and 2003 | -At least one antibiotic prescription throughout pregnancy till date of birth according to medical registers  -Prevalence: 23.8%  Timing of exposure during pregnancy had varying effects for both outcome types. | -Up to 5 years  -Two outcomes: asthma hospitalization and asthma medication  (Also eczema, non-significant relationship, but no tables to know what co-variates had been examined) | Any prenatal antibiotics:  -Asthma hospitalization:  HR:1.17 (1.00-1.36)  -Asthma medication: HR:1.18 (1.10-1.27)  Only prenatal non-respiratory antibiotics:  -Asthma hospitalization: HR:1.32 (1.12-1.56)  -Asthma medication: HR:1.23 (1.10-1.37) | *Dose-response*  *relationship* | Yes | Selection: 4/4  Comparability:1/2  Outcome:2/3  Total:7 (medium) |
|  |  |  |  |  |  | *Intra-partum*  *Antibiotics* | unclear |  |
|  |  |  |  |  |  | *Types of antibiotics* | In a sample of mothers who had taken antibiotics for non-respiratory infections, effect was stronger than for any antibiotics (both significant), especially in mothers without asthma history |  |
|  |  |  |  |  |  | *Child postnatal*  *antibiotic usage* | Not examined |  |
|  |  |  |  |  |  | *Gender* | Adjusted for |  |
|  |  |  |  |  |  | *Birth weight* | Adjusted for |  |
|  |  |  |  |  |  | *Delivery mode* | Adjusted for |  |
|  |  |  |  |  |  | *Family allergies* | Relationship stronger in children of mothers without asthma history |  |
|  |  |  |  |  |  | *Breastfeeding* | Not examined |  |
|  |  |  |  |  |  | *Maternal infections* | Infections not examined, but only antibiotics for certain indications |  |
|  |  |  |  |  |  | *Childhood infections* | Not examined |  |
| **Martel, 2009**^45^  Case-control study  *Increased risks of childhood asthma were found for several predictors, including*  *prescription of antibiotics within the first 6 months of life, male gender, asthma during pregnancy, use of antibiotics during pregnancy, paternal asthma, and asthma in siblings.* | Canada | 5226 cases and 104,520 controls for first stage of study.  Study period: 1990-2002 | No. of antibiotic prescriptions throughout pregnancy (unclear if this includes intra-partum antibiotics) | -Up to 10 years  -Prevalence 32.6% with asthmatic mothers and 14.1% with non-asthmatic mothers | OR: 1.15 (1.03-1.29) | *Dose-response*  *relationship* | Yes | Selection: 4/4  Comparability:1/2  Exposure:3/3  Total: 8 (low) |
|  |  |  |  |  |  | *Intra-partum*  *Antibiotics* | Unclear |  |
|  |  |  |  |  |  | *Types of antibiotics* | Not examined |  |
|  |  |  |  |  |  | *Child postnatal*  *antibiotic usage* | At least one antibiotic prescription predictor of asthma (besides prenatal antibiotic exposure) |  |
|  |  |  |  |  |  | *Gender* | Male gender independent predictor |  |
|  |  |  |  |  |  | *Birth weight* | Only Small for Gestations Age (SGA) examined, but not a predictor |  |
|  |  |  |  |  |  | *Delivery mode* | Planned CS predictor in model only including maternal/ pregnancy characteristics, the significance disappeared after adding postnatal factors. |  |
|  |  |  |  |  |  | *Family allergies* | Maternal asthma, paternal asthma and sibling asthma each independent predictors |  |
|  |  |  |  |  |  | *Breastfeeding* | Independently protective |  |
|  |  |  |  |  |  | *Maternal infections* | Not examined |  |
|  |  |  |  |  |  | *Childhood infections* | At least one diagnosis of bronchopulmonary disease (wheezing, bronchitis, bronchiolitis, pneumonia) was an independent predictor. |  |

**Table S2b**. Prenatal antibiotic exposure and childhood eczema: study characteristics and findings

S: study * CS: caesarean section * RC: reverse causation * OR: odds ratio * HR: hazard ration * RR: relative risk * NOS: Newcastle-Ottawa Scale (8-9 low risk; 6-7 medium risk; <6 unclear)

| **Study/ authors’ conclusions (relevant for this review)** | **Country** | **Population** | **-Timing antibiotic**  **exposure**  **-Prevalence of**  **exposure** | **Health outcome, age and prevalence** | **Summary measures (HR, RR or OR)** | **Influential factors** | | **Risk of bias**  **Using the**  **NOS**  8-9: low risk  6-7: medium risk <6: unclear risk |
| --- | --- | --- | --- | --- | --- | --- | --- | --- |
|  | | | | | | | | |
| **Timm, 2016**^46^  Population cohort study  *Prenatal exposure to antibiotics throughout*  *pregnancy was associated with an increased risk of atopic dermatitis but only within the first 18 months of life among children born by atopic mothers.* | Denmark | 62 560 mother-child pairs  Recruited from 1996-2002 | -Antibiotic use throughout pregnancy obtained by means of interviews at 30 weeks of pregnancy and 6 months after birth.  -Prevalence: 20.3% | -Eczema  -Infants 18 months  -Prevalence: 16% | Antibiotic exposure only in 1^st^/2^nd^ or only in 3^rd^ trimester insignificant for babies with atopic and non-atopic mothers .  Atopic mothers + exposure in 1^st^ to 2^nd^ and 3^rd^ trimesters:  OR 1.45 (1.19-1.76)    Non-atopic mothers + exposure in 1st to 2^nd^ and 3^rd^ trimesters:  OR 1.01 (0.83-1.22) | *Dose-response*  *relationship* | Not examined | Selection: 4/4  Comparability:1/2  Outcome:2/3  Total:7 (medium) |
|  |  |  |  |  |  | *Intra-partum*  *Antibiotics* | Unclear |  |
|  |  |  |  |  |  | *Types of antibiotics* | No differences seen in antibiotic types |  |
|  |  |  |  |  |  | *Child postnatal*  *antibiotic usage* | Not examined |  |
|  |  |  |  |  |  | *Gender* | Not examined |  |
|  |  |  |  |  |  | *Birth weight* | Not examined |  |
|  |  |  |  |  |  | *Delivery mode* | In the group of children with atopic mothers who had had antibiotic exposure throughout pregnancy (in two categorizations), the effect size of those with CS was higher (OR 2.23 versus 1.34, both significant) |  |
|  |  |  |  |  |  | *Family allergies* | Only children with atopic mothers were at risk |  |
|  |  |  |  |  |  | Breastfeeding | Not examined |  |
|  |  |  |  |  |  | Maternal infections | Not examined |  |
|  |  |  |  |  |  | Childhood infections | Not examined |  |
| **Wohl, 2015**^47^  Retrospective cohort study  *Only when intra-partum antibiotic exposure lasts >24 hours, it is significantly associated with eczema* | United States | 492 mother-child pairs  Born from 1996-2008 | -Intra-partum exposure  -Prevalence: 26% | -Up to 2 years of age  -Prevalence: 27.8% | RR 1.03 (0.75-1.41)  >24 hours exposure:  RR 1.99 (1.13-3.49) | *Dose-response relationship* | Relationship only significant when exposure lasted 24+ hours | Selection: 3/4  Comparability:0/2  Outcome:2/3  Total: 5 (Unclear) |
|  |  |  |  |  |  | *Intra-partum*  *Antibiotics* | Yes, the exposure is only intra-partum antibiotics |  |
|  |  |  |  |  |  | *Types of antibiotics* | Univariably examined, no differences |  |
|  |  |  |  |  |  | *Child postnatal*  *antibiotic usage* | Not examined |  |
|  |  |  |  |  |  | *Gender* | Univariably examined, no differences |  |
|  |  |  |  |  |  | *Birth weight* | Univariably examined, effect size birth weight <2500 substantially higher, but not significant |  |
|  |  |  |  |  |  | *Delivery mode* | Sample only consisted of vaginal births |  |
|  |  |  |  |  |  | *Family allergies* | Univariably examined, no differences according to parental or sibling alllergies |  |
|  |  |  |  |  |  | *Breastfeeding* | Univariably examined, no differences |  |
|  |  |  |  |  |  | *Maternal infections* | Not examined |  |
|  |  |  |  |  |  | *Childhood infections* | Not examined |  |
| **Dom, 2010**^48^  Prospective birth cohort study  *While antibiotic administration to children up to 1 year of age was insignificantly protective, and antibiotics at >1 year was significantly protective, prenatal antibiotic exposure significantly increased the risk of eczema.* | Belgium | 773 children  Pregnant women recruited from 1997-2001 | -Prenatal antibiotic usage by means of questionnaires at 5 months pregnancy and 3 months after birth. (Also examined postnatal antibiotics through breastfeeding and medication)  -Prevalence: 19.9% | -Up to 4 years  -Prevalence: 36.3% | OR 1.82 (1.14-2.92) | *Dose-response*  *relationship* | Not examined | Selection: 4/4  Comparability: 1/2  Outcome:1/3  Total: 6 (medium) |
|  |  |  |  |  |  | *Intra-partum*  *Antibiotics* | Unclear |  |
|  |  |  |  |  |  | *Types of antibiotics* | Not examined |  |
|  |  |  |  |  |  | *Child postnatal*  *antibiotic usage* | Adjusted for |  |
|  |  |  |  |  |  | *Gender* | Adjusted for |  |
|  |  |  |  |  |  | *Birth weight* | Adjusted for |  |
|  |  |  |  |  |  | *Delivery mode* | Not examined |  |
|  |  |  |  |  |  | *Family allergies* | Adjusted for parental allergies |  |
|  |  |  |  |  |  | *Breastfeeding* | Adjusted for |  |
|  |  |  |  |  |  | *Maternal infections* | Not examined |  |
|  |  |  |  |  |  | *Childhood infections* | Not examined |  |

**Table S3a**. Infant antibiotic administration and childhood asthma

S: study * CS: caesarean section * RC/CbI: Reverse causation/Confounding by indication * NOS: Newcastle-Ottawa Scale (8-9 low risk; 6-7 medium risk; <6 unclear) * HR: hazard ratio * OR: odds ratio * RR: relative risk

| **Study & authors’ conclusions (relevant for this review)** | **Country** | **Population** | **-Timing of antibiotic**  **exposure**  **-Prevalence exposure** | **-Health outcome/age -Prevalence outcome** | | **Summary measures (HR, RR or OR)** | **Influential factors** | | | **RC/CbI and Risk of bias using the NOS** ^31^  8-9: low risk  6-7:medium risk  <6: unclear risk |
| --- | --- | --- | --- | --- | --- | --- | --- | --- | --- | --- |
| **Asthma** | | | | | | | | | | |
| **Yoshida, 2018**^35^  Retrospective cohort studies  **S1**: main study  **S2**: sibling matched  *Exposure to antibiotics during the first year of life was associated with childhood asthma even after adjusting for familial factors. The association of prenatal exposure with asthma was weaker, but still significant.* | Japan | **S1**: 83470 children  **S2**: 28085 children  Born between 2005-2014 | Prenatal exposure (see table 1a) and antibiotic administration during 1^st^ year  -------------------  **S1**: Prevalence 56%  **S2**: Prevalence 57% | Asthma  Age 1 - 6 years  --------------------  **S1**: Prevalence 4.4%  **S2**: Prevalence 5.6% | **S1**: Age of onset:  1-< 3 years: HR 2.43 (2.20-2.69)  3-<6 years: HR: 1.23 (1.11-1.36)  **S2**:Age of onset 1-6 years: HR 1.62 (1.27-2.07) | | | *Dose-response*  *relationship* | Yes, in both studies | RC and/or CbI considered:No  Selection: 3/4  Comparability:1/2  Outcome:2/3  Total:6 (medium) |
|  |  |  |  |  |  |  |  | *Types of antibiotics* | In both studies, macrolides and cephalosporins were significant |  |
|  |  |  |  |  |  |  |  | *Prenatal antibiotics* | Not examined |  |
|  |  |  |  |  |  |  |  | *Gender* | Univariably examined, male gender at greater risk |  |
|  |  |  |  |  |  |  |  | *Birth weight* | Not examined |  |
|  |  |  |  |  |  |  |  | *Delivery mode* | Not examined |  |
|  |  |  |  |  |  |  |  | *Family allergies* | S1: no adjustment for familial asthma. S2: Adjustments for siblings |  |
|  |  |  |  |  |  |  |  | *Breastfeeding* | Not examined |  |
|  |  |  |  |  |  |  |  | *Childhood Infections* | Not examined |  |
| **Ahmadizar, 2017**^49^  S1: Prospective cohort in the Netherlands  S2: Prospective cohort in Scotland  *Both cohorts in the Netherlands and Scotland show that children treated with antibiotics in the first 3 years of life are more likely to develop asthma.* | The Nether-lands/  Scotland | S1: 7393 children  S2: 891 children whose pregnant mothers were recruited between 1997 and 1999 | S1: Exposure first 3 years of age  Prevalence 1^st^ year: 55%  S2: Exposure first 6 months  Prevalence: 29% | **S1**: Any asthma up to 9 years of age  **S2**: Any asthma up to 10 years of age  --------------------  **S1**: Prevalence 10%  **S2**: Prevalence 8% | S1: Exposure 0-3 years  OR 2.84 (1.70-4.75)  Exposure 1^st^ year  OR 3.21 (1.89-5.45)  Exposure 2^nd^ year  OR 2.25 (1.18-4.30)  Exposure 3^rd^ year  OR 2.21 (0.92-5.33)  Higher effects at lower age of antibiotics exposure  S2: Exposure first 6 months: OR 1.50, 95% CI: 0.91-2.46) | | | *Dose-response*  *relationship* | Not examined | RC and/or CbI considered:No  Selection: 3/4  Comparability:1/2  Outcome :3/3  Total:7 (medium) |
|  |  |  |  |  |  |  |  | *Types of antibiotics* | Not examined |  |
|  |  |  |  |  |  |  |  | *Prenatal antibiotics* | Not examined |  |
|  |  |  |  |  |  |  |  | *Gender* | Adjusted for agenda |  |
|  |  |  |  |  |  |  |  | *Birth weight* | Not examined |  |
|  |  |  |  |  |  |  |  | *Delivery mode* | Not examined |  |
|  |  |  |  |  |  |  |  | *Family allergies* | history of asthma/ allergies (both studies) |  |
|  |  |  |  |  |  |  |  | *Breastfeeding* | Not examined |  |
|  |  |  |  |  |  |  |  | *Childhood infections* | Not examined |  |
| **Stromberg Celind, 2017**^50^  Prospective cohort study  *Antibiotic treatment during the first week of life was associated with an increased risk of atopic asthma at 12 years, suggesting an immune-mediated effect.* | Sweden | 3637 children  Born in 2003 | Exposure 1^st^ week after birth  Prevalence not reported | Current asthma  12 years of age  Prevalence: 6.4% (65% atopic and 35% non-atopic)  Cumulative prevalence up to 12 years: 14% | Atopic asthma:  OR 2.2 (1.2–4.2)  Current asthma: OR 1.9 (1.1-3.2)  Non-atopic asthma: OR 1.4 (0.5-3.4) | | | *Dose-response*  *relationship* | Not examined | RC and/or CbI considered:Yes  (reverse causation unlikely as exposure was measured during 1st week of life)  Selection: 4/4  Comparability:1/2  Outcome :3/3  Total:8 (low) |
|  |  |  |  |  |  |  |  | *Types of antibiotics* | Not examined, but mentioned that broad-spectrum antibiotics are always given during 1^st^ week |  |
|  |  |  |  |  |  |  |  | *Prenatal antibiotics* | Maternal medication included in multivariable model, not significant. |  |
|  |  |  |  |  |  |  |  | *Gender* | Male gender significant predictor of atopic, not non-atopic asthma |  |
|  |  |  |  |  |  |  |  | *Birth weight* | Not examined |  |
|  |  |  |  |  |  |  |  | *Delivery mode* | Examined as potential confounder, not included in multivariable model |  |
|  |  |  |  |  |  |  |  | *Family allergies* | Parental asthma predictive for both types of asthma |  |
|  |  |  |  |  |  |  |  | *Breastfeeding* | Breastfeeding 4+ months significantly protective for non-atopic asthma, but not for atopic or any asthma |  |
|  |  |  |  |  |  |  |  | *Childhood infections* | Not examined, but childhood eczema was also a predictor |  |
| **Eldeirawi, 2014**^51^  Cross-sectional study  Their findings suggest that antibiotic use in infancy is associated with childhood asthma, but the associations are limited to subgroups of children (i.e. those who did not have ear infections in first year) | USA | 2023 Mexican American children  Study period:  2004-2005 | Exposure during first year  Prevalence: 40% | Age of outcome: 4-18 years  Prevalence doctor-diagnosed asthma: 7.4% | OR 2.33 (1.53-3.55)  Significance is driven by children without ear infections in first year: OR 2.37 (1.39-4.02)  Sample of children with ear infections, excluding those diagnosed with asthma in first year: OR 0.97 (0.47-1.98) | | | *Dose-response*  *relationship* | Yes | RC and/or CbI considered:Yes  Selection: 3/4  Comparability:1/2  Outcome: 2/3  Total:6 (medium) |
|  |  |  |  |  |  |  |  | *Types of antibiotics* | Not examined |  |
|  |  |  |  |  |  |  |  | *Prenatal antibiotics* | Not examined |  |
|  |  |  |  |  |  |  |  | *Gender* | Univariably examined, males significantly more at risk, therefore adjusted for in main analyses |  |
|  |  |  |  |  |  |  |  | *Birth weight* | Not examined |  |
|  |  |  |  |  |  |  |  | *Delivery mode* | Not examined |  |
|  |  |  |  |  |  |  |  | *Family allergies* | Univariably examined, parental history of asthma or allergies significant, therefore adjusted for in main analyses |  |
|  |  |  |  |  |  |  |  | *Breastfeeding* | Not examined |  |
|  |  |  |  |  |  |  |  | *Childhood infections* | Univariably examined, ear infections were significant, therefore adjusted for in main analyses. Other significant associations examined univariably were wheeze, rhinitis and dry cough at night. |  |
| **Metsälä, 2015**^42^  Case-control study  *Both prenatal and post-natal exposure to antibiotics was associated with an increased risk of asthma.* | Finland | 6690 case-control pairs  Born from 1996-2004 | Prenatal and exposure during first year of life | Asthma  Age 3-9 years (mean 4.3) | OR = 1.60 (1.48–1.73)  Diagnosed between ages 3-5 OR = 1.68 (1.54–1.85)  Diagnosed at the age of 6-9 years OR 1.33 (1.12–1.58)  Effect size is higher at younger age of onset | | | *Dose-response*  *relationship* | Yes | RC and/or CbI considered:Yes  RC: Antibiotics purchased in previous 6 months excluded to avoid RC.  Also looked at onset after 6 years, to address RC  Selection: 4/4  Comparability:1/2  Exposure:2/3  Total:7 (medium) |
|  |  |  |  |  |  |  |  | *Types of antibiotics* | Cephalosporins,  sulphonamides and trimethoprim,  macrolides and amoxicillin significant. |  |
|  |  |  |  |  |  |  |  | *Prenatal antibiotics* | Examined separately, also examined in postnatal model, but excluded due to not altering findings in the effects of postnatal antibiotics. |  |
|  |  |  |  |  |  |  |  | *Gender* | Univariably examined, male gender significant |  |
|  |  |  |  |  |  |  |  | *Birth weight* | Not examined |  |
|  |  |  |  |  |  |  |  | *Delivery mode* | Univariably examined, delivery mode significant |  |
|  |  |  |  |  |  |  |  | *Family allergies* | Adjusted for maternal asthma |  |
|  |  |  |  |  |  |  |  | *Breastfeeding* | Not examined |  |
|  |  |  |  |  |  |  |  | *Childhood infections* | Only antibiotics for certain infectious diseases examined. Antibiotics used for gram-positive or respiratory tract infections were significant, antibiotics for urinary tract infections had an effect, but were insignificant. Antibiotics used for both had the greatest significant effect. |  |
| **Lee, 2015**^52^  Cross-sectional study  *Bronchiolitis in the first 2 years of life, antibiotic exposure during the first year of life, and parental history of asthma were independent risk factors for the development of asthma. When combined, antibiotic*  *use and a history of* *bronchiolitis increased the risk of asthma (adjusted odds ratio [aOR]: 4.64, 95% confidence interval [CI]: 3.09-6.97)* | South Korea | 7,389 middle school students  Study period: 2008, 2011 | Exposure (3+ days)in the 1^st^ year of life  Prevalence: 30.3% | Mean age: 13.9 years  Asthma ever: 7.77%  Current asthma: 1.74% | Exposure 3+ days: OR 1.94 (1.49-2.53)  History of bronchiolitis in first two years  OR 3.27 ( 2.32-4.60)  Combined effect:  OR 4.64 ( 3.09-6.97) | | | *Dose-response*  *relationship* | Yes | RC and/or CbI considered:Yes  Selection: 3/4  Comparability:1/2  Outcome:2/3  Total:6 (medium) |
|  |  |  |  |  |  |  |  | *Types of antibiotics* | Not examined |  |
|  |  |  |  |  |  |  |  | *Prenatal antibiotics* | Not examined |  |
|  |  |  |  |  |  |  |  | *Gender* | Female gender significantly protective |  |
|  |  |  |  |  |  |  |  | *Birth weight* | Not examined |  |
|  |  |  |  |  |  |  |  | *Delivery mode* | Not significant in multivariable model |  |
|  |  |  |  |  |  |  |  | *Family allergies* | Parental history of asthma/ other allergies significant in multivariable model |  |
|  |  |  |  |  |  |  |  | *Breastfeeding* | Not significant in multivariable model |  |
|  |  |  |  |  |  |  |  | *Childhood infections* | Bronchiolitis independently significant in multivariable model, combination of bronchiolitis and antibiotics increased effect (OR 4.64(3.09-6.97) |  |
| **Pitter, 2015**^53^  Population cohort study  *Antibiotic consumption in the first year of life increase the risk of new-onset asthma with a significant dose–response relationship (also after adjusting for respiratory infections)* | Italy | 143,163 children  Born in the period 1995–2011 | Exposure 1^st^ year of life  Prevalence not reported | Asthma  From 13 months to 18 years of age  Incidence: 24.4% | IRR: 1.50 (1.46–1.53)  The risk was highest for asthma identified at 13–35 months of life compared to later periods, although all were significant | | | *Dose-response*  *relationship* | Yes | RC and/or CbI considered:Yes  Selection: 4/4  Comparability:1/2  Outcome: 3/3  Total:8 (low) |
|  |  |  |  |  |  |  |  | *Types of antibiotics* | All types significant, strongest effects in macrolides, penicillins and cephalosporins |  |
|  |  |  |  |  |  |  |  | *Prenatal antibiotics* | Not examined |  |
|  |  |  |  |  |  |  |  | *Gender* | Invariably examined, male gender significant, adjusted for in main analyses |  |
|  |  |  |  |  |  |  |  | *Birth weight* | Univariably examined, children with low birth weight <2500 significant, adjusted for in main analyses |  |
|  |  |  |  |  |  |  |  | *Delivery mode* | Univariably examined, children with CS significant |  |
|  |  |  |  |  |  |  |  | *Family allergies* | Not examined |  |
|  |  |  |  |  |  |  |  | *Breastfeeding* | Not examined |  |
|  |  |  |  |  |  |  |  | *Childhood infections* | Univariably examined, respiratory infections significant and adjusted for in main analyses |  |
| **Krenz-Niedbała, 2015**^54^  Cross-sectional study  *In the relationship between breastfeeding duration and asthma risk, a mediating role was played by antibiotic use during the first year of the child’s life.*  *(i.e. longer breastfeeding led to lower antibiotic usage, which led to lower risk of asthma)* | Poland | 1277 children  Children born in 1999 | Exposure: breastfeeding duration (months)  -Antibiotics examined as possible mediator  Antibiotic use during first 12 months: prevalence 53.1% | Asthma  8 years old  Prevalence:  10.2% | Antibiotics and asthma  OR 1.71 (1.29-2.26)  Antibiotics accounts for 24% of the relationship between breastfeeding and asthma | | | *Dose-response*  *relationship* | Not examined | RC and/or CbI considered:No  Selection: 3/4  Comparability:1/2  Outcome:2/3  Total:6 (medium) |
|  |  |  |  |  |  |  |  | *Types of antibiotics* | Not examined |  |
|  |  |  |  |  |  |  |  | *Prenatal antibiotics* | Not examined |  |
|  |  |  |  |  |  |  |  | *Gender* | Removed during backward regression, so not in final model |  |
|  |  |  |  |  |  |  |  | *Birth weight* | Not examined |  |
|  |  |  |  |  |  |  |  | *Delivery mode* | Not examined |  |
|  |  |  |  |  |  |  |  | *Family allergies* | Not examined |  |
|  |  |  |  |  |  |  |  | *Breastfeeding* | Duration significantly protective for asthma, but partially mediated (by 24%) by antibiotic use in first year |  |
|  |  |  |  |  |  |  |  | *Childhood infections* | Not examined |  |
| **Khalkhali, 2014**^55^  Case control study  *The findings suggest that antibiotic consumption in children is associated with*  *an increased risk of childhood asthma.* | Iran | Doctor-diagnosed asthma cases: 207  Controls (children without respiratory symptoms): 400  Conducted between March and September 2010 | Exposure during first year of life  Antibiotic exposure Cases:71.9%  Controls: 53% | Asthma age of outcome: 2-8 years | OR 1.91 (1.27-2.88) | | | *Dose-response*  *relationship* | Not examined | RC and/or CbI considered:No  Selection: 3/4  Comparability:1/2  Exposure:2/3  Total: 6 (medium) |
|  |  |  |  |  |  |  |  | *Types of antibiotics* | Not examined |  |
|  |  |  |  |  |  |  |  | *Prenatal antibiotics* | Only examined univariably (no effect size reported), but due to insignificance not included in main model |  |
|  |  |  |  |  |  |  |  | *Gender* | Univariably no significant differences |  |
|  |  |  |  |  |  |  |  | *Birth weight* | Univariably no significant differences |  |
|  |  |  |  |  |  |  |  | *Delivery mode* | CS univariably significant, but in multivariable model insignificant |  |
|  |  |  |  |  |  |  |  | *Family allergies* | Adjusted for family history of asthma/atopic disorder |  |
|  |  |  |  |  |  |  |  | *Breastfeeding* | Not examined |  |
|  |  |  |  |  |  |  |  | *Childhood Infections* | Not examined |  |
| **Ortqvist, 2014**^43^  S1: Prospective cohort study  S2: Sibling case-control study  *Positive associations between exposure to antibiotics in fetal and early life and subsequent childhood asthma could be caused by confounding due to shared familial factors and to respiratory infections*. | Sweden | S1: 493 785 children  S2: 180 894    Born from 2006-2010 | Exposure from 0-2+ years  Prevalence all children exposed to antibiotics: 62% | Asthma:  Up to school age (unclear what age is meant)  Prevalence in cohort: 6%  The lower the age of antibiotic exposure, the stronger the effect | Higher significant effect the lower the age of antibiotic exposure  S1: 0-0.5 years (antibiotic exposure). HR 3.71 (3.41 - 4.03)  1-1.5 years: HR 2.31 (2.20 – 2.43)  2+ years: HR 1.80 (1.68 1.93)  S2: Sibling analyses:  0-0.5 years: HR 2.11 (1.61 to 2.76)  1-1.5 years: HR 1.82 (1.55 to 2.13)  2+ years: HR 1.32 (0.97 to 1.79)  Antibiotics first year and asthma from age 2 onwards:  S1: HR 1.23 (1.15 to 1.32)  S2: HR 0.90 (0.66 to 1.23) | | | *Dose-response*  *relationship* | Yes | RC and/or CbI considered:Yes  Selection:4 /4  Comparability:1/2  Outcome:2/3  Total: 7 (medium) |
|  |  |  |  |  |  |  |  | *Types of antibiotics* | S1: Antibiotics for respiratory tract infections (including amoxicillin, penicillin, cephalosporin and macrolides) much stronger effects than antibiotics for urinary/skin tract infections (including trimethoprim and sulphonamide), although both significant (HR 4.12 versus 1.54)  S2: Antibiotics for respiratory tract infections lower significant effect, and antibiotics for urinary/skin infections insignificant |  |
|  |  |  |  |  |  |  |  | *Prenatal antibiotics* | Adjusted for |  |
|  |  |  |  |  |  |  |  | *Gender* | Adjusted for |  |
|  |  |  |  |  |  |  |  | *Birth weight* | Adjusted for |  |
|  |  |  |  |  |  |  |  | *Delivery mode* | Adjusted for |  |
|  |  |  |  |  |  |  |  | *Family allergies* | 2^nd^ study adjusted for siblings |  |
|  |  |  |  |  |  |  |  | *Breastfeeding* | Not examined |  |
|  |  |  |  |  |  |  |  | *Childhood infections* | Not examined, but antibiotics for different indications examined. |  |
| **Goksör, 2013**^56^  Prospective cohort study  *The early introduction of fish and neonatal antibiotic treatment influence the risk of asthma into school age.* | Sweden | 4051 children born in 2003 | Exposure: only broad-spectrum antibiotics given during 1^st^ week of life  Prevalence: 4.6% | 8 years old  Prevalence: 5.7% | Current asthma OR 2.3 ( 1.2–4.2).  Significance is driven by association with atopic asthma (i.e. having concurrent allergic sensitization and /or other diagnosed food allergies or eczema). Association is insignificant for non-atopic asthma.  Only significant if asthma onset was before preschool age:  OR 3.0 ( 1.5–6.0)  Compared to onset after preschool age: OR 1.05 (0.4–  2.7)  Stronger effect seen in younger versus older age groups | | | *Dose-response*  *relationship* | Not examined | RC and/or CbI considered:Yes  Selection: 4/4  Comparability:1/2  Outcome: 3/3  Total: 8 (low) |
|  |  |  |  |  |  |  |  | *Types of antibiotics* | Not examined |  |
|  |  |  |  |  |  |  |  | *Prenatal antibiotics* | Adjusted for maternal medication (not defined) ,but not significant in multivariable model |  |
|  |  |  |  |  |  |  |  | *Gender* | Male gender independent risk factor |  |
|  |  |  |  |  |  |  |  | *Birth weight* | Not examined |  |
|  |  |  |  |  |  |  |  | *Delivery mode* | Included in multivariable model, but not significant |  |
|  |  |  |  |  |  |  |  | *Family allergies* | Parental asthma, eczema or rhinoconjunctivitis independent risk factor for asthma. When asthma types were differentiated, only significant for non-atopic asthma. |  |
|  |  |  |  |  |  |  |  | *Breastfeeding* | Breastfeeding 4+ months included in multivariable model, but not significant |  |
|  |  |  |  |  |  |  |  | *Childhood infections* | Not examined, but eczema and food allergy are also independent risk factors. |  |
| **Almqvist, 2012**^57^  Prospective cohort study  *Their findings suggest that the association between*  *antibiotics and asthma is subject to either reverse causation or confounding by indication due to respiratory tract infections. The association was significant for antibiotics to treat Gram-positive infections such as respiratory tract infections, but not for antibiotics to treat urinary tract or skin and soft tissue infections.* | Sweden | 211 192 children who received at least one antibiotic prescription between 2005 and 2009  Born from 2005-2009 | Exposure first 3 years  Prevalence: 100% had been exposed to at least one antibiotic at some point | 0-4 years  Asthma prescription  Prevalence: 45% had taken asthma medication | Age of outcome:  <1 year HR 2.20 (2.12–2.28)  1 year HR 1.73 (1.66–1.82)  2 years HR 1.29 (1.15–1.45)  3+ years HR 1.15 (0.73–1.83)  The effect is stronger in the younger age of onset groups | | | *Dose-response*  *relationship* | Yes | RC and/or CbI considered:Yes  Selection: 4/4  Comparability:1/2  Outcome :2/3  Total: 7 (medium) |
|  |  |  |  |  |  |  |  | *Types of antibiotics* | -Effects stronger for  amoxicillin, penicillin, cephalosporin and macrolides (for Gram-positive infections) than sulphonamides, trimethoprim and  quinolones (for urinary tract infections) and flucloxacillin (skin and soft tissue infections).  -Stronger effect seen in broad-spectrum compared to narrow-spectrum antibiotics. |  |
|  |  |  |  |  |  |  |  | *Prenatal antibiotics* | Not examined |  |
|  |  |  |  |  |  |  |  | *Gender* | Analyses stratified according to gender; both showed significant effects, but female gender had somewhat higher effect sizes |  |
|  |  |  |  |  |  |  |  | *Birth weight* | Not examined |  |
|  |  |  |  |  |  |  |  | *Delivery mode* | Not examined |  |
|  |  |  |  |  |  |  |  | *Family allergies* | Not examined |  |
|  |  |  |  |  |  |  |  | *Breastfeeding* | Not examined |  |
|  |  |  |  |  |  |  |  | *Childhood infections* | Antibiotics used as proxies for infection types. |  |
| **Muc, 2013**^58^  Cross-sectional study  *There is a significant association between the exposures paracetamol and antibiotics and the outcomes prevalence and severity of asthma* | Portugal | 1,037 children  School children recruited from 2011 -2012 | Exposure 1^st^ year of life  Prevalence: 23.1% | Current asthma and asthma ever  Age 6-9 years (mean 7.2)  Asthma ever prevalence: 10.4% | Current asthma: OR 1.6(1.0–2.5), asthma ever: OR 2.0 (1.3–3.1) | | | *Dose-response*  *relationship* | Not examined | RC and/or CbI considered:No  Selection: 3/4  Comparability:1/2  Outcome:1/3  Total:5 (unclear) |
|  |  |  |  |  |  |  |  | *Types of antibiotics* | Not examined |  |
|  |  |  |  |  |  |  |  | *Prenatal antibiotics* | Not examined |  |
|  |  |  |  |  |  |  |  | *Gender* | Adjusted for |  |
|  |  |  |  |  |  |  |  | *Birth weight* | Not examined |  |
|  |  |  |  |  |  |  |  | *Delivery mode* | Not examined |  |
|  |  |  |  |  |  |  |  | *Family allergies* | Adjusted for family history of asthma and rhinitis |  |
|  |  |  |  |  |  |  |  | *Breastfeeding* | Not examined |  |
|  |  |  |  |  |  |  |  | *Childhood infections* | Not examined |  |
| **Jedrychowski, 2011**^59^  Prospective cohort study  *Any antibiotics significantly associated with asthma, until adjusted for no. of respiratory infections. Broad-spectrum antibiotics (macrolides and cephalosporins) remain significant after adjusting for respiratory infections, providing evidence for a causal relationship.* | Poland | 310 children  Pregnant women recruited from 2001-2004 | Any exposure in previous 6 months  During whole exposure period till 5 years: 78.8% | Asthma  5 years  Prevalence: 9.3% | Any antibiotics OR 1.65  (0.93 – 2.93)  Significance found only with macrolides: OR 2.14 (1.16-3.95) and cephalosporins: OR 1.98 (1.14-3.37) | | | *Dose-response*  *relationship* | Yes, seen with macrolides and cephalosporins | RC and/or CbI considered:Yes  Selection: 3/4  Comparability:1/2  Outcome :1/3  Total:5 (unclear) |
|  |  |  |  |  |  |  |  | *Types of antibiotics* | Only macrolides and cephalosporins were significant |  |
|  |  |  |  |  |  |  |  | *Prenatal antibiotics* | Not examined |  |
|  |  |  |  |  |  |  |  | *Gender* | Univariably examined, incidence higher in boys, but not significant |  |
|  |  |  |  |  |  |  |  | *Birth weight* | Univariably examined, no significant differences |  |
|  |  |  |  |  |  |  |  | *Delivery mode* | Not examined |  |
|  |  |  |  |  |  |  |  | *Family allergies* | Adjusted for maternal atopy |  |
|  |  |  |  |  |  |  |  | *Breastfeeding* | Not examined |  |
|  |  |  |  |  |  |  |  | *Childhood infections* | Adjusted for no.of respiratory tract infections |  |
| **Risnes, 2010**^60^  Prospective cohort study  *Authors conclude that antibiotic exposure before 6 months of age is associated with asthma and allergy at 6 years of age, particularly in children without family history of asthma; protopathic bias is unlikely to account for the main findings* | United States | 1,401 children  Many children also included who had mothers with asthma.  Study recruitment of pregnant women: 1997-2000 | 0-6 months  Prevalence: 33.1% | Asthma  6 years  Prevalence: 11.7% | Asthma  OR 1.52 (1.07-2.16) | | | *Dose-response relationship* | Yes, effect is significant starting from 2+ courses. | RC and/or CbI considered:Yes  Selection: 3/4  Comparability:1/2  Outcome:1/3  Total:5 (unclear) |
|  |  |  |  |  |  |  |  | *Types of antibiotics* | Not examined |  |
|  |  |  |  |  |  |  |  | *Prenatal antibiotics* | Not examined |  |
|  |  |  |  |  |  |  |  | *Gender* | Univariably examined, but no differences |  |
|  |  |  |  |  |  |  |  | *Birth weight* | Univariably examined, but no differences |  |
|  |  |  |  |  |  |  |  | *Delivery mode* | Univariably examined, but no differences |  |
|  |  |  |  |  |  |  |  | *Family allergies* | Univariably examined, having siblings with asthma is significant.  Effect stronger within sample of children without parental asthma than those with parental asthma. |  |
|  |  |  |  |  |  |  |  | *Breastfeeding* | Adjusted for |  |
|  |  |  |  |  |  |  |  | *Childhood infections* | Lower respiratory tract infections (LRTI) univariably examined, significantly associated with asthma. Effect of antibiotics was stronger in children who had not had LRTI in first year than those who did have LRTI in first year. |  |
| **Su, 2010**^61^  Prospective cohort study  *The relation of asthma to antibiotics in this cohort appears to be an artefact of the strong relation of number of physician visits for illness with both antibiotic use and risk for*  *asthma.* | United States | 424 children  Pregnant women recruited between 1997 and 2003 | Exposure in the 1^st^ 9 months of life  Prevalence: 32.1% | Ever asthma and eczema up to 5 years | Relationship insignificant  OR: 1.2 [0.6, 2.3] Dose-response relationship is significant without including respiratory infections, and insignificant when including no. of physician visits.  No. of physician visits significant OR 1.5 [1.1–1.9]) | | | *Dose-response*  *relationship* | Yes | RC and/or CbI considered:Yes  Selection: 4/4  Comparability:1/2  Outcome:2/3  Total: 7 (medium) |
|  |  |  |  |  |  |  |  | *Types of antibiotics* | Not examined |  |
|  |  |  |  |  |  |  |  | *Prenatal antibiotics* | Not examined |  |
|  |  |  |  |  |  |  |  | *Gender* | Univariably examined, males are significant, adjusted for in main analyses |  |
|  |  |  |  |  |  |  |  | *Birth weight* | Not examined |  |
|  |  |  |  |  |  |  |  | *Delivery mode* | No examined |  |
|  |  |  |  |  |  |  |  | *Family allergies* | Maternal asthma univariably examined, but not significant |  |
|  |  |  |  |  |  |  |  | *Breastfeeding* | Breastfeeding for at least 3 months adjusted for |  |
|  |  |  |  |  |  |  |  | *Childhood infections* | Adjusted for no. of visits |  |
| **Marra, 2009**^62^  Prospective cohort study  *Antibiotic exposure in the first year of life was associated with a small risk of developing asthma in early childhood* | Canada | 251817 children  From birth cohort between 1997 and 2003 | Exposure 1^st^ year of life  Prevalence: 43% | Asthma  2-9 years old:  Prevalence: 7% | HR: 1.12 (1.08–1.16) | | | *Dose-response*  *relationship* | Clear dose-response relationship, greatest at >4 doses | RC and/or CbI considered:Yes  Reverse causation addressed by excluding children with possible asthma symptoms during first two years.- When children with URTI, LRTI, Bronchitis, or Otitis Media were excluded, there was a somewhat stronger significant effect.  Selection: 4/4  Comparability:1/2  Outcome:2/3  Total:7 (medium) |
|  |  |  |  |  |  |  |  | *Types of antibiotics* | Macrolides had the strongest exposure effect, but cephalosporin, penicillin and amoxicillin also had significant effects. |  |
|  |  |  |  |  |  |  |  | *Prenatal antibiotics* | Not examined |  |
|  |  |  |  |  |  |  |  | *Gender* | Males independent risk factor |  |
|  |  |  |  |  |  |  |  | *Birth weight* | Not examined |  |
|  |  |  |  |  |  |  |  | *Delivery mode* | CS independent risk factor |  |
|  |  |  |  |  |  |  |  | *Family allergies* | Not examined |  |
|  |  |  |  |  |  |  |  | *Breastfeeding* | Not examined |  |
|  |  |  |  |  |  |  |  | *Childhood infections* | Adjusted for physician visits (also independent risk factor), allergist/ respirologist/ immunologist visits, and hospital visits involving  surgery, congenital anomalies, and related diseases (otitis media (also significantly protective), bronchitis, URTI, and LRTI) (also independent risk factors)). |  |
| **Martel, 2009**^45^  Case-control study  *A range of significant predictors of asthma (up to the age of 10) found including prescription of antibiotics within the first 6 months of life, male gender, asthma during pregnancy, use of antibiotics during pregnancy and at least 1 previous diagnosis of bronchopulmonary disease* | Canada | For final multivariable model  Cases: 745 children  Controls: 873 children  Born from 1990-2002 | Exposure 1^st^ 6 months after birth  Prevalence: 27% | Asthma up to 10 years old | OR 1.70 (1.34, 2.15) | | | *Dose-response*  *relationship* | Not examined | RC and/or CbI considered:Yes  Selection: 4/4  Comparability:1/2  Exposure:3/3  Total:8 (low) |
|  |  |  |  |  |  |  |  | *Types of antibiotics* | Not examined |  |
|  |  |  |  |  |  |  |  | *Prenatal antibiotics* | No. of prenatal antibiotic prescriptive independent  risk factor |  |
|  |  |  |  |  |  |  |  | *Gender* | Male gender independent risk factor |  |
|  |  |  |  |  |  |  |  | *Birth weight* | Not examined |  |
|  |  |  |  |  |  |  |  | *Delivery mode* | Not significant in multivariable model |  |
|  |  |  |  |  |  |  |  | *Family allergies* | Maternal asthma, paternal asthma and sibling asthma each independent risk factors |  |
|  |  |  |  |  |  |  |  | *Breastfeeding* | Breastfeeding independently protective |  |
|  |  |  |  |  |  |  |  | *Childhood infections* | At least one diagnosis of bronchopulmonary disease (wheezing, bronchitis, bronchiolitis, pneumonia) also risk factors. |  |
| **Garcia, 2008**^63^  Cross-sectional study  *Factors associated with current asthma symptoms among the 6–7 year age-group included higher maternal education, a cat in the home watching TV 1–2 hours/day and medication with acetaminophen in the first and most recent year of life or antibiotics in the first year of life .*  *Antibiotics not significant predictor in model examining 13-14 year olds.* | Colombia | 3256 children in the 6–7 age group  3829 children in the  13–14 age group  Study period: 2002 | Exposure 1^st^ year | Age of outcome:6-7 years and 13-14 years  Current asthma symptoms for both groups respectively: 10.4% versus 8.6% | Current asthma: 6-7 years  OR 1.9 (1.4–2.5)  It correlated strongly with acetaminophen use in 1^st^ year, and was therefore excluded in a final model  (not shown)  Current asthma: 13-14 years  No relationship seen univariably, therefore not included in multivariable model | | | *Dose-response*  *relationship* | Not examined | RC and/or CbI considered:No  Selection: 3/4  Comparability:1/2  Outcome:1/3  Total:5 (unclear) |
|  |  |  |  |  |  |  |  | *Types of antibiotics* | Not examined |  |
|  |  |  |  |  |  |  |  | *Prenatal antibiotics* | Not examined |  |
|  |  |  |  |  |  |  |  | *Gender* | Univariably examined, not eligible for multivariable model |  |
|  |  |  |  |  |  |  |  | *Birth weight* | Not examined |  |
|  |  |  |  |  |  |  |  | *Delivery mode* | Univariably examined, not eligible for multivariable model |  |
|  |  |  |  |  |  |  |  | *Family allergies* | Not examined |  |
|  |  |  |  |  |  |  |  | *Breastfeeding* | Univariably examined, not eligible for multivariable model |  |
|  |  |  |  |  |  |  |  | *Childhood infections* | Not examined |  |

**Table S3b.** Infant antibiotic administration and childhood eczema

S: study * CS: caesarean section * RC/CbI: Reverse causation/Confounding by indication * NOS: Newcastle-Ottawa Scale (8-9 low risk; 6-7 medium risk; <6 unclear) * HR: hazard ratio * OR: odds ratio * RR: relative risk

| **Study/ authors’ conclusions (relevant for this review)** | **Country** | **Population** | **-Timing of antibiotic**  **exposure**  **-Prevalence exposure** | **Health outcome/age and prevalence** | **Summary measures (HR, RR or OR)** | | **Influential factors** | | | **RV/CbI and**  **Risk of bias**  **Using the**  **NOS**  8-9: low risk  6-7:medium risk  <6: unclear risk |
| --- | --- | --- | --- | --- | --- | --- | --- | --- | --- | --- |
| Eczema | | | | | | | | | | |
| **Oosterloo, 2017**^72^  Prospective cohort study  *Antibiotic treatment in the first week of life is associated with an increased risk of wheezing and infantile colics, but not eczema in first year* | The Nether-  lands | 436 newborn infants staying in one of four teaching hospitals for more than 24 hours  Born from 2012 - 2015 | Exposure during first week of life  Prevalence: 34.6% | Up to 1 year old  Various outcomes including eczema  Parental reported eczema: 35.8 %  Doctor reported eczema:  13.3 % | | Parental reported  OR 1.09 (0.7-1.7)  Doctor reported  OR 0.85 (0.45-1.61) | | *Dose-response*  *relationship* | Only antibiotic duration measured (2-3 days versus 7 days), no significant differences | RC and/or CbI considered:Yes  (reverse causation unlikely as exposure is measured in 1^st^ week of life)  Selection: 4/4  Comparability:1/2  Outcome:2/3  Total:7 (medium) |
|  |  |  |  |  |  |  |  | *Types of antibiotics* | All the infants received broad-spectrum antibiotics. Differences between antibiotic types not examined |  |
|  |  |  |  |  |  |  |  | *Prenatal antibiotics* | Univariably examined, maternal antibiotic use was significant, but not included in multivariable model. |  |
|  |  |  |  |  |  |  |  | *Gender* | Not examined |  |
|  |  |  |  |  |  |  |  | *Birth weight* | Univariably examined,not significant |  |
|  |  |  |  |  |  |  |  | *Delivery mode* | Adjusted for |  |
|  |  |  |  |  |  |  |  | *Family allergies* | Adjusted for parental atopic disease |  |
|  |  |  |  |  |  |  |  | *Breastfeeding* | Adjusted for breastfeeding duration |  |
|  |  |  |  |  |  |  |  | *Childhood infections* | Not examined |  |
| **Park, 2016**^73^  Cross-sectional study  Other factors were significantly associated with atopic dermatitis, such as parental allergic diseases, but not antibiotic use during infancy | South Korea | 4003 children of 6-7 years  4112 children of 12-13 years | Antibiotic use during infancy (specific period of time not reported)  Prevalence: 42.8% (sample 6-7 years)  Prevalence: 31.5 (12-13 years) | 6-7 years:  AD diagnosis ever: 35.6%  12-13 years  AD diagnosis ever: 24.2% | | AD diagnosis ever:  6-7 years:  OR 1.09 (0.88-1.39)  13-14 years:  OR 1.04 (0.86-1.34)  Univariably examined: significant, multivariably examined insignificant | | *Dose-response*  *relationship* | Not examined | RC and/or CbI considered:No  Selection: 3/4  Comparability:1/2  Outcome:2/3  Total: 6 (medium) |
|  |  |  |  |  |  |  |  | *Types of antibiotics* | Not examined |  |
|  |  |  |  |  |  |  |  | *Prenatal antibiotics* | Not examined |  |
|  |  |  |  |  |  |  |  | *Gender* | 6-7 years: Univariably examined, not significant.  12-13 years: Female in multivariable model. |  |
|  |  |  |  |  |  |  |  | *Birth weight* | Not examined |  |
|  |  |  |  |  |  |  |  | *Delivery mode* | Univariably examined, not significant both age groups |  |
|  |  |  |  |  |  |  |  | *Family allergies* | Maternal and paternal allergy each significant for both age groups |  |
|  |  |  |  |  |  |  |  | *Breastfeeding* | Univariably examined, not significant both age groups |  |
|  |  |  |  |  |  |  |  | *Childhood infections* | Not examined |  |
| **Taylor-Robinson, 2016**^74^  Prospective cohort study  *Antibiotic exposure in the first year of life is one of a set of factors, including maternal atopy, breastfeeding (1–6 weeks and ≥ 6 months), introduction of solids under 4 months or cow’s milk under 9 months associated with eczema by age.* | United Kingdom | 11 537 children  Children born between 2000 and 2002 | Exposure in the first year of life | Ever eczema  Up to age 5  Prevalence: 35.1% | | OR 1.28 (1.16-1.42) | | *Dose-response*  *relationship* | Not examined | RC and/or CbI considered:No  Selection: 3/4  Comparability:1/2  Outcome:2/3  Total: 6 (medium) |
|  |  |  |  |  |  |  |  | *Types of antibiotics* | Not examined |  |
|  |  |  |  |  |  |  |  | *Prenatal antibiotics* | Not examined |  |
|  |  |  |  |  |  |  |  | *Gender* | Female gender significant risk factor in multivariable model |  |
|  |  |  |  |  |  |  |  | *Birth weight* | Not significant in multivariable model |  |
|  |  |  |  |  |  |  |  | *Delivery mode* | Not significant in multivariable model |  |
|  |  |  |  |  |  |  |  | *Family allergies* | Maternal asthma and/or eczema |  |
|  |  |  |  |  |  |  |  | *Breastfeeding* | Breastfeeding 1-6 weeks and >6 months significant risk factor (<1 week and 6 weeks to 6 months not significant) |  |
|  |  |  |  |  |  |  |  | *Childhood infections* | Not examined |  |
| **Loo, 2015**^75^  Prospective cohort study  *Early-onset AD was mainly associated with familial factors, while late-onset AD was associated with the consumption of antibiotics or probiotics from 9-10 months.* | Singapore | 792 children | Exposure first 6 months | Doctor-diagnosed eczema: 23.6%  < 6 months: 53,5%  6-12 months: 23.0%  12-18 months: 25.1% | | Only significant when diagnosed at 12-18 months  OR 3.11 ( 1.10–8.76)  Diagnosis at 6-12 months: OR 3.19 (0.72-14.11) | | *Dose-response*  *relationship* | Not examined | RC and/or CbI considered: No  Selection: 4/4  Comparability:1/2  Outcome:1/2  Total:6 (medium) |
|  |  |  |  |  |  |  |  | *Types of antibiotics* | Not examined |  |
|  |  |  |  |  |  |  |  | *Prenatal antibiotics* | Not examined |  |
|  |  |  |  |  |  |  |  | *Gender* | Female gender |  |
|  |  |  |  |  |  |  |  | *Birth weight* | Not significant in multivariable model |  |
|  |  |  |  |  |  |  |  | *Delivery mode* | Not significant in multivariable model |  |
|  |  |  |  |  |  |  |  | *Family allergies* | Maternal allergy, but not paternally allergy significant risk factor |  |
|  |  |  |  |  |  |  |  | *Breastfeeding* | Not examined |  |
|  |  |  |  |  |  |  |  | *Childhood infections* | Not examined |  |
| **Garcia-Marcos, 2010**^76^  Cross-sectional study  *Early exposure to paracetamol or to antibiotics is independently associated with an increased prevalence of eczema at school age. Asthma and/or rhinoconjunctivitis substantially modifies this association: that of paracetamol disappears and that of antibiotics is increased, when compared to children without respiratory symptoms.* | Spain | 13,908 children  Study period | Exposure in 1^st^ year of life  Prevalence: 49.5% | Eczema  Ages 6-7 years  Prevalence: 6.9% | | Eczema OR 1.52 (1.30–1.77) | | *Dose-response*  *relationship* | Not examined | RC and/or CbI considered:No  Selection: 3/4  Comparability:1/2  Outcome: 1/3  Total: 5 (unclear) |
|  |  |  |  |  |  |  |  | *Types of antibiotics* | Not examined |  |
|  |  |  |  |  |  |  |  | *Prenatal antibiotics* | Not examined |  |
|  |  |  |  |  |  |  |  | *Gender* | Adjusted for |  |
|  |  |  |  |  |  |  |  | *Birth weight* | Not examined |  |
|  |  |  |  |  |  |  |  | *Delivery mode* | Not examined |  |
|  |  |  |  |  |  |  |  | *Family allergies* | Not examined |  |
|  |  |  |  |  |  |  |  | *Breastfeeding* | Not examined |  |
|  |  |  |  |  |  |  |  | *Childhood infections* | Not examined |  |
| **Dom, 2010**^48^  (various outcomes including eczema, also examined prenatal antibiotics)  *Although prenatal exposure was associated with an increased risk for eczema, exposure through lactation and medication in first year was not. Exposure through medication after first year was protective however, for eczema*. | Belgium | 70 children (with data on eczema)  Pregnant women recruited from 1997-2001 | Prenatal and exposure during 1^st^ year of life (indirect exposure during lactation and through medication)  Prevalence: 67.5%    During 1^st^ three months of lactation: 8.4% | Up to 4 years  Eczema ever  Prevalence: 36.3% | | Eczema  Through breastfeeding  OR 1.14 (0.56–2.35)  Through medication (exposed in the 1^st^ year)  OR 0.61 (0.36–1.01)  Through medication (exposed after the 1^st^ year)  OR 0.11 (0.05–0.23) | | *Dose-response*  *relationship* | Not examined | RC and/or CbI considered:No  RC: although authors mention that their study being prospective prevents reverse causation  Selection: 4/4  Comparability:1/2  Outcome:1/3  Total:6 (medium) |
|  |  |  |  |  |  |  |  | *Types of antibiotics* | Not examined |  |
|  |  |  |  |  |  |  |  | *Prenatal antibiotics* | Adjusted for |  |
|  |  |  |  |  |  |  |  | *Gender* | Adjusted for |  |
|  |  |  |  |  |  |  |  | *Birth weight* | Adjusted for |  |
|  |  |  |  |  |  |  |  | *Delivery mode* | Not examined |  |
|  |  |  |  |  |  |  |  | *Family allergies* | Adjusted for parental allergic allergy |  |
|  |  |  |  |  |  |  |  | *Breastfeeding* | Adjusted for |  |
|  |  |  |  |  |  |  |  | *Childhood infections* | Only adjusted for LRTI in examination of wheezing as outcome, not eczema. |  |
| **Schmitt, 2009**^77^  Prospective cohort study  *Crude analyses suggested that early infections and exposure to antibiotics are risk factors for AE. However, stratified analyses indicated that early infections were only associated with a higher rate of AE when treated with broad-spectrum antibiotics such as cephalosporines or macrolides.*  *Antibiotic treatment appears to modify the association between early infections and subsequent AE.* | Germany | 370 children with no eczema in 1^st^ year of life | Exposure during 1^st^ year of life  Prevalence: 41.9%  Aim was to determine whether antibiotics modified relationship between infections and eczema | Atopic eczema in 2^nd^ year of life | | RR 1.66 (0.95 - 2.90)  2+ courses: RR 2.11 (1.05-4.22) | | *Dose-response*  *relationship* | Yes | RC and/or CbI considered:Yes  Small number of children with eczema: 44  Selection:4 /4  Comparability:1/2  Outcome:2/3  Total:7 (medium) |
|  |  |  |  |  |  |  |  | *Types of antibiotics* | Only infections treated with cephalosporins and macrolides are significant |  |
|  |  |  |  |  |  |  |  | *Prenatal antibiotics* | Not examined |  |
|  |  |  |  |  |  |  |  | *Gender* | More girls than boys with eczema (27 versus 17), but not statistically examined |  |
|  |  |  |  |  |  |  |  | *Birth weight* | Not examined |  |
|  |  |  |  |  |  |  |  | *Delivery mode* | Not examined |  |
|  |  |  |  |  |  |  |  | *Family allergies* | Not examined |  |
|  |  |  |  |  |  |  |  | *Breastfeeding* | Not examined |  |
|  |  |  |  |  |  |  |  | *Childhood infections* | Infections were associated with eczema in general; infections not treated with antibiotics were insignificant, only infections treated with cephalosporins and macrolides were usually significant. |  |

**Table S3c.** Infant antibiotic administration and childhood hay fever

S: study * CS: caesarean section * RC/CbI: Reverse causation/Confounding by indication * NOS: Newcastle-Ottawa Scale (8-9 low risk; 6-7 medium risk; <6 unclear) * HR: hazard ratio * OR: odds ratio * RR: relative risk

| **Study/ authors’ conclusions (relevant for this review)** | **Country** | | **Population** | **-Timing of antibiotic**  **exposure**  **-Prevalence exposure** | **Health outcome/age and prevalence** | **Summary measures (HR, RR or OR)** | **Influential factors** | | **RV/CbI and**  **Risk of bias**  **Using the**  **NOS** ^31^  8-9: low risk  6-7: medium risk <7: unclear risk |
| --- | --- | --- | --- | --- | --- | --- | --- | --- | --- |
| Hay fever | | | | | | | | | |
| **Wang X, 2016**^78^  (allergic rhinitis)  Cross-sectional study  *After adjustment by age, sex, family history of atopy, and respondent of questionnaire, they find that no siblings, mother of older age during pregnancy, shorter breastfeeding, using antibiotics in the first year, and home dampness-related exposures, had significant associations with increased prevalence of the studied diseases.* | | China | 13,335 children  Study period: 2011-2012 | Exposure 1^st^ year  Prevalence unknown | Age of outcome: 4-6 years  Prevalences: 12.6% (doctor-diagnosed), 54.1% (rhinitis ever), and 42.7% (rhinitis symptoms past year) | Allergic rhinitis ever  OR1.23 (1.09–1.40) | *Dose-response*  *relationship* | Not examined | RC and/or CbI considered:No  Selection: 3/4  Comparability: 1/2  Outcome: 2/3  Total:6 (medium) |
|  |  |  |  |  |  |  | *Types of antibiotics* | Not examined |  |
|  |  |  |  |  |  |  | *Prenatal antibiotics* | Not examined |  |
|  |  |  |  |  |  |  | *Gender* | Adjusted for |  |
|  |  |  |  |  |  |  | *Birth weight* | Not examined |  |
|  |  |  |  |  |  |  | *Delivery mode* | Not examined |  |
|  |  |  |  |  |  |  | *Family allergies* | Adjusted for family history of atopy |  |
|  |  |  |  |  |  |  | *Breastfeeding* | Shorter exclusive breastfeeding significant risk factor |  |
|  |  |  |  |  |  |  | *Childhood infections* | Not examined |  |
| **Tamay,2014**^79^  Cross-sectional study  *Frequent paracetamol and antibiotic use in the first year of life, history of frequent upper respiratory tract infections, adenotonsillectomy,*  *breastfeeding less than six months, dog at home or perianal redness in the first year of life, and frequent trucks passing near the home were independent risk factors of allergic rhinitis at 6-7 years of age.* | | Turkey | 9,875 children  Study period 2004-2005 | Exposure first year of life  Prevalence not mentioned | Age of outcome: 6-7 years  Prevalences: 44.3% (lifetime), 29.2% (current) and 8.1% (physician-diagnosed) | Physician –diagnosed allergic rhinitis  OR 1.41 (1.15-1.73) | *Dose-response*  *relationship* | Not examined | RC and/or CbI considered:Yes  Selection: 3/4  Comparability:1/2  Outcome:1/3  Total:5 (unclear) |
|  |  |  |  |  |  |  | *Types of antibiotics* | Not examined |  |
|  |  |  |  |  |  |  | *Prenatal antibiotics* | Not examined |  |
|  |  |  |  |  |  |  | *Gender* | Univariably examined, not significant |  |
|  |  |  |  |  |  |  | *Birth weight* | Not examined |  |
|  |  |  |  |  |  |  | *Delivery mode* | Not examined |  |
|  |  |  |  |  |  |  | *Family allergies* | Not examinec |  |
|  |  |  |  |  |  |  | *Breastfeeding* | Breastfeeding <6 months significant risk factor |  |
|  |  |  |  |  |  |  | *Childhood infections* | Upper respiratory tract infections significant risk factor |  |
| **Alm, 2014**^80^  Prospective cohort study  *Factors including antibiotics in the first week of life, eczema in first year, doctor diagnosed food allergy increased the risk of allergic rhinitis at school age, while living on a farm at preschool age reduced the risk.* | | Sweden | 4033 children born in 2003 | Exposure: first week of life  Prevalence: 4.7& | Hay fever  8 years old  Prevalence: 14.0 % | OR 1.75 (1.03, 2.97) | *Dose-response relationship* | Not examined | RC and/or CbI considered:No  Selection:4/4  Comparability:1/2  Outcome:2/3  Total:7 (medium) |
|  |  |  |  |  |  |  | *Types of antibiotics* | Not examined |  |
|  |  |  |  |  |  |  | *Prenatal antibiotics* | Not examined |  |
|  |  |  |  |  |  |  | *Gender* | Male gender significant risk factor |  |
|  |  |  |  |  |  |  | *Birth weight* | Not examined |  |
|  |  |  |  |  |  |  | *Delivery mode* | Univariably insignificant, so  not included in multivariable model |  |
|  |  |  |  |  |  |  | *Family allergies* | Parental hay fever significant risk factor |  |
|  |  |  |  |  |  |  | *Breastfeeding* | Univariably not significant, so not included in multivariable model |  |
|  |  |  |  |  |  |  | *Childhood infections* | Not examined |  |
| **Peñaranda, 2012**^81^  Cross-sectional study  *Factors associated with self-reported allergic rhinitis among children included current asthma and atopic dermatitis symptoms; use of acetaminophen in the first year of life and in the last 12 months; antibiotic use in the first year of life; high school and university maternal education; smokers at home; and caesarean delivery.*  *Antibiotic use in the 1st year was not one of the predictors of asthma in adolescents.* | | Colombia | 3,256 children aged 6 – 7  3,830 adolescents aged 13 - 14 years  Study period: 2002 | Exposure 1^st^ year of life  Prevalence not reported | Hay fever children  Prevalence: 30.8%  Adolescents  Prevalence: 36.6% | Hay fever in 6-7 year olds  OR 1.3 (1.1-1.7)  Hay fever in 13-14 year olds  Not mentioned, but not part of multivariable model due to its insignificance. | *Dose-response*  *relationship* | Not examined | RC and/or CbI considered:No  Selection:3/4  Comparability:1/2  Outcome:1/3  Total: 5 (unclear) |
|  |  |  |  |  |  |  | *Types of antibiotics* | Not examined |  |
|  |  |  |  |  |  |  | *Prenatal antibiotics* | Not examined |  |
|  |  |  |  |  |  |  | *Gender* | No significant difference |  |
|  |  |  |  |  |  |  | *Birth weight* | Univariably examined, little effect so not included in multivariable model |  |
|  |  |  |  |  |  |  | *Delivery mode* | CS significant risk factor in 6-7 years old in multivariable model |  |
|  |  |  |  |  |  |  | *Family allergies* | Not examined |  |
|  |  |  |  |  |  |  | *Breastfeeding* | Not examined |  |
|  |  |  |  |  |  |  | *Childhood infections* | Not examined |  |

**Table S3d.** Infant antibiotic administration and childhood allergies (publications examining multiple allergies)

S: study * CS: caesarean section * RC/CbI: Reverse causation/Confounding by indication * NOS: Newcastle-Ottawa Scale (8-9 low risk; 6-7 medium risk; <6 unclear) * HR: hazard ratio * OR: odds ratio * RR: relative risk

| **Study/ authors’ conclusions (relevant for this review)** | **Country** | | **Population** | **-Timing of antibiotic**  **exposure**  **-Prevalence exposure** | **Health outcome/age and prevalence** | **Summary measures (HR, RR or OR)** | **Influential factors** | | **RV/CbI and**  **Risk of bias**  **Using the**  **NOS**^31^  8-9: low risk  6-7:medium risk  <6: unclear risk |
| --- | --- | --- | --- | --- | --- | --- | --- | --- | --- |
| Studies focusing on multiple allergies including asthma, eczema and hay fever | | | | | | | | | |
| **Mitre, 2018**^64^  Retrospective cohort study  *This study found associations between the use of acid-suppressive medications and antibiotics during the first 6 months of infancy and subsequent development of allergic disease.* | | United States | 792130 children  Born from 2001-2013 | Exposure 1^st^ 6 months after birth  Prevalence: 16% | Various allergies from 6 months till 5 years of age onwards  Prevalences:  Asthma: 14,1%  Hay fever: 31.3%  Eczema: 14.2% | Asthma  HR2.09 (2.05-2.13)  Hay fever  HR 1.75 (1.72-1.78)  Eczema  HR 1.18 (1.16-1.19) | *Dose-response*  *relationship* | Not examined | RC and/or CbI considered:No  Selection: 4/4  Comparability:1/2  Outcome: 1/3  Total: 6 (medium) |
|  |  |  |  |  |  |  | *Types of antibiotics* | Not examined |  |
|  |  |  |  |  |  |  | *Prenatal antibiotics* | Not examined |  |
|  |  |  |  |  |  |  | *Gender* | Males significantly more likely to have all three allergies |  |
|  |  |  |  |  |  |  | *Birth weight* | Not examined |  |
|  |  |  |  |  |  |  | *Delivery mode* | CS significant for all three allergies |  |
|  |  |  |  |  |  |  | *Family allergies* | Not examined |  |
|  |  |  |  |  |  |  | *Breastfeeding* | Not examined |  |
|  |  |  |  |  |  |  | *Childhood infections* | Not examined |  |
| **Yamamoto-Hanada, 2017**^65^  (asthma, hay fever, eczema)  Prospective cohort study  *The findings suggest that antibiotic use within the first 2 years of life was a risk factor for current asthma, current atopic dermatitis, and current allergic rhinitis in 5-year-old children* | | Japan | 1196 children  Born from 2004-2006 | Exposure first 2 years of life  Prevalence: 48.3% | Age 5  Current asthma  Prevalence: 10.5%  Current eczema  Prevalence: 21.5%  Current hay fever  Prevalence: 10.6% | -Current asthma  OR 1.72 (1.10,2.70)  -Current eczema  OR 1.40 ( 1.01,1.94)  -Current hay fever  OR 1.65 ( 1. 05, 2.58) | *Dose-response*  *relationship* | Not examined | RC and/or CbI considered:Yes  Selection: 3/4  Comparability:2/2  Outcome:3/3  Total:8 (low) |
|  |  |  |  |  |  |  | *Types of antibiotics* | Significance driven by cephems (cephalosporins) for asthma and hay fever and macrolides for hay fever |  |
|  |  |  |  |  |  |  | *Prenatal antibiotics* | Not examined |  |
|  |  |  |  |  |  |  | *Gender* | Adjusted for |  |
|  |  |  |  |  |  |  | *Birth weight* | Not examined |  |
|  |  |  |  |  |  |  | *Delivery mode* | Adjusted for |  |
|  |  |  |  |  |  |  | *Family allergies* | Adjusted for maternal history of allergy |  |
|  |  |  |  |  |  |  | *Breastfeeding* | Not examined |  |
|  |  |  |  |  |  |  | *Childhood infections* | Adjusted for bronchitis |  |
| **Wang, 2013**^66^  Two prospective cohort studies  S1: Born in 1998  S2: Born in 2003  *There was a positive relationship between acetaminophen, as well as antibiotic exposure during the 1st year of life and the subsequent development of the three examined allergic diseases (atopic dermatitis, asthma and allergic rhinitis) in the 1998 birth cohort, but this effect in the 2003 cohort, especially for atopic dermatitis and asthma, was lower and not statistically significant.* | | Taiwan | -S1: 263 620  children born in 1998  -S2: 9910 children born in 2003 | Exposure to antibiotics in first year after birth  Prevalence S1: 87%  Prevalence S2: 49% | Ages 2-6 years  S1: 1998:  Asthma: 21.7%  Hay fever: 31.2%  Eczema: 7.2%  S2: 2003:  Asthma: 28.0%  Hay fever: 37.2%  Eczema: 10.0% | S1:1998 only antibiotics  Eczema: HR 1.61 (1.53–1.70)  Asthma: HR 1.38 (1.32–1.46)  Hay fever: 1.41 (1.35–1.47)  S2:2003 only antibiotics  Eczema: 1.03 (0.73–1.44)  Asthma : 0.96 (0.72–1.29)  Hay fever: 1.25 (0.99–1.59) | *Dose-response*  *relationship* | Only seen in 1998 cohort for all allergies | RC and/or CbI considered:Yes  Children who developed allergic  diseases during the first 2 years of life were excluded in the subsequent analyses.  Selection: 4/4  Comparability:1/2  Outcome: 2/3  Total:7 (medium) |
|  |  |  |  |  |  |  | *Types of antibiotics* | Not examined |  |
|  |  |  |  |  |  |  | *Prenatal antibiotics* | Not examined |  |
|  |  |  |  |  |  |  | *Gender* | Male gender significant for all three allergies |  |
|  |  |  |  |  |  |  | *Birth weight* | Not examined |  |
|  |  |  |  |  |  |  | *Delivery mode* | Not examined |  |
|  |  |  |  |  |  |  | *Family allergies* | Not examined |  |
|  |  |  |  |  |  |  | *Breastfeeding* | Not examined |  |
|  |  |  |  |  |  |  | *Childhood infections* | -Subsamples according to respiratory tract infections showed that in the 1998 cohort of children, antibiotics were only significant for all three allergies in children without respiratory tract infections. In the 2003 cohort, antibiotics were only significant for eczema and hay fever in children without respiratory tract infections.  -Univariable analyses show health care utilization (incl. of otitis media diagnoses) to be significantly associated with eczema. |  |
| **Hoskin-Parr, 2013**^67^  Prospective population-based cohort study  *A dose-response relationship was found between antibiotic use in the first 2 yr of life and asthma at age 7.5 yr. Reverse causation may explain part of the association, as this effect was reduced after excluding wheezing from 0-18* *months. Excluding wheezing from 0-30 months was reduced to insignificance, (although still dose-response effect)* | | United Kingdom | 5780 children Avon Longitudinal Study of Parents and Children (ALSPAC)  Born from 1991-1992 | Antibiotic use in first 2 years of life reported by mothers  0-6 months: 30.9%  6-15 months: 53.3%  15-24 months: 46%  Only significant in period 6-15 months. Strongest effect when exposure was in all three periods. | 7.5 years  Prevalences:  Asthma: 10.7%  Eczema: 17.2%  Hay fever: 8.7% | Any antibiotic exposure  Asthma:  OR 1.75 (1.40-2.17)  Hay fever:  OR 1.28 (1.03-1.60)  Eczema:  OR 1.20 (1.02–1.41)  Excluding wheezing from 0-30 months led to insignificance. | *Dose-response*  *relationship* | Hay fever: only 4+ courses significant. Asthma: clear dose-response relationship, significant from 2+ courses | RC and/or CbI considered:Yes  RC addressed by excluding children with wheezing first from 0-18 months, then from 0-30 months.  Selection: 4/4  Comparability:1/2  Outcome: 3/3  Total:8 (low) |
|  |  |  |  |  |  |  | *Types of antibiotics* | Not examined |  |
|  |  |  |  |  |  |  | *Prenatal antibiotics* | Not examined |  |
|  |  |  |  |  |  |  | *Gender* | Boys significantly more likely to have asthma and hay fever and less likely to have eczema |  |
|  |  |  |  |  |  |  | *Birth weight* | Low birth weight significantly associated with asthma |  |
|  |  |  |  |  |  |  | *Delivery mode* | In multivariable model, no significant effects |  |
|  |  |  |  |  |  |  | *Family allergies* | Not examined |  |
|  |  |  |  |  |  |  | *Breastfeeding* | Not examined |  |
|  |  |  |  |  |  |  | *Childhood*  *Infections* | Not examined |  |
| **Mai, 2010**^68^  Prospective cohort study  *After adjustment for respiratory infections during the first year of life, only the associations with wheeze and asthma (not eczema) at age 4 years remained statistically significant. At age 8 years,*  *antibiotic use during the first year of life was significantly associated with wheeze and eczema after adjustment for early life factors, the effects becoming lower when*  *adjusting for the respiratory infections. The association between early antibiotic use and later*  *allergic disease could at least partially be explained by early respiratory infection.* | | Sweden | 3306 children  Born from 1994-1996 | Exposure in 1st year of life  Prevalence: 43% | Age of outcome: prevalence at 4 years and 8 years respectively  Asthma 7% and 6%  Allergic rhinitis 11% and 14%  Eczema 20% and 16% | 4 years  Asthma  OR 1.4 (1.1–1.7)  Allergic rhinitis  OR 1.0 (0.9–1.3)  Eczema  OR 1.0 (0.9–1.2)    8 years  Asthma  OR 1.2 (0.9–1.5)  Allergic rhinitis  OR 1.0 (0.8–1.2)  Eczema  OR 1.0 (0.9–1.2) | *Dose-response*  *relationship* | 4 years: only seen for asthma, but not for hay fever  8 years: no dose-response relationships | RC and/or CbI considered:Yes  Selection: 4/4  Comparability:1/2  Outcome:3/3  Total:8 (low) |
|  |  |  |  |  |  |  | *Types of antibiotics* | Not examined |  |
|  |  |  |  |  |  |  | *Prenatal antibiotics* | Not examined |  |
|  |  |  |  |  |  |  | *Gender* | Adjusted for |  |
|  |  |  |  |  |  |  | *Birth weight* | Not examined |  |
|  |  |  |  |  |  |  | *Delivery mode* | Not examined |  |
|  |  |  |  |  |  |  | *Family allergies* | Adjusted for parental allergy |  |
|  |  |  |  |  |  |  | *Breastfeeding* | Adjusted for exclusive breastfeeding |  |
|  |  |  |  |  |  |  | *Childhood infections* | Adjusted for |  |
| **Foliaki, 2009**^69^  (study covers children from 20 countries)  Cross-sectional study  *There is an association between antibiotic use*  *in the first year of life and current symptoms of asthma,* *rhinoconjunctivitis, and eczema in children 6 and 7 years old. Further research is required to determine whether the observed associations are causal or are a result of confounding by indication or reverse causation.* | | New Zealand  Data from 20 countries | 103,653 children | Exposure during the first year  Prevalence not mentioned | Ages 6-7 years  Various outcomes including current asthma and eczema.  Prevalences not mentioned | Asthma ever:  OR 1.94 (1.83-2.06)  Current severe wheeze:  1.82 (1.67-1.98)  Current symptoms of eczema:  OR 1.42 (1.33-1.51) | *Dose-response relationship* | Not examined | RC and/or CbI considered:No  Selection: 3/4  Comparability:1/2  Outcome:2/3  Total:6 (medium) |
|  |  |  |  |  |  |  | *Types of antibiotics* | Not examined |  |
|  |  |  |  |  |  |  | *Prenatal antibiotics* | Not examined |  |
|  |  |  |  |  |  |  | *Gender* | Adjusted for |  |
|  |  |  |  |  |  |  | *Birth weight* | Not examined |  |
|  |  |  |  |  |  |  | *Delivery mode* | Not examined |  |
|  |  |  |  |  |  |  | *Family allergies* | Not examined |  |
|  |  |  |  |  |  |  | *Breastfeeding* | Adjusted for |  |
|  |  |  |  |  |  |  | *Childhood infections* | Not examined |  |
| **Wickens, 2008**^70^  Prospective cohort study  *Much of the effects of antibiotics found are due to the effects of chest infection. Antibiotic exposure is strongly associated with no. of chest infections. The association between antibiotics and asthma is complex. There is some indication that antibiotics may have an effect on eczema at 15 months.* | | New Zealand | Mothers recruited between 1997 and 2001 | Antibiotic exposure <3 mths  Prevalence: 14.1%  Antibiotic exposure <15 mths  Prevalence: 72.1% | Current asthma  15 months  Prevalence: 11.8%  3-4 years  Prevalence: 17.4%  Eczema:  15 months: 39.6%  3-4 years: 31.9% | **Asthma**  Exp. <3 mths, outcome: <15 mths  OR 1.27 (0.75–2.17) (adjusted for chest infections)  Exp. <15 mths, outcome: 3-4 years  OR 0.78 (0.46–1.32)  **Eczema**  Exp. <3 mths, outcome: <15 mths  OR 0.69 (0.47–1.03)  Exp. <15 mths, outcome: 3-4 years  OR 1.52 (0.87–2.65) | *Dose-response relationship* | Not examined | RC and/or CbI considered:Yes  Selection: 4/4  Comparability: 1/2  Outcome: 2/3  Total:7 (medium) |
|  |  |  |  |  |  |  | *Types of antibiotics* | Higher effects and significance only with broad spectrum antibiotics |  |
|  |  |  |  |  |  |  | *Prenatal antibiotics* | Not examined |  |
|  |  |  |  |  |  |  | *Gender* | Adjusted for |  |
|  |  |  |  |  |  |  | *Birth weight* | Not examined |  |
|  |  |  |  |  |  |  | *Delivery mode* | Not examined |  |
|  |  |  |  |  |  |  | *Family allergies* | Adjusted for parental asthma |  |
|  |  |  |  |  |  |  | *Breastfeeding* | Not examined |  |
|  |  |  |  |  |  |  | *Childhood infections* | Adjusted for chest infections. Exposure before 3 months and asthma onset by 15 months only significant in children who had had one or more chest infections. |  |
| **Kusel, 2008**^71^  Prospective cohort study  *Their findings did find any indication that antibiotic use in early life led to subsequent asthma or atopy at 5 years.* | | Australia | 198 children (all with at least one parent with a doctor diagnosis of asthma, hay fever or eczema) | Exposure 1^st^ year of life  Prevalence: 54% | Age up to 5 years  Prevalence ever eczema: 66.2%  Current eczema at 4-5 years: 31.3%  Diagnosed asthma: 28.8%  Current asthma: 18.7% | Current eczema: OR 0.9 (0.4-2.3)  Doctor diagnosis of asthma:  OR 1.5 (0.7-3.2) | *Dose-response*  *relationship* | Examined, but no dose response relationship seen | RC and/or CbI considered:Yes    Selection: 3/4  Comparability:1/2  Outcome:3/3  Total:7 (medium) |
|  |  |  |  |  |  |  | *Types of antibiotics* | Not examined |  |
|  |  |  |  |  |  |  | *Prenatal antibiotics* | Not examined |  |
|  |  |  |  |  |  |  | *Gender* | Adjusted for, also no significant differences seen |  |
|  |  |  |  |  |  |  | *Birth weight* | Not examined |  |
|  |  |  |  |  |  |  | *Delivery mode* | Not examined |  |
|  |  |  |  |  |  |  | *Family allergies* | Adjusted for antibiotic propensity score which included parental histories of asthma and atopy |  |
|  |  |  |  |  |  |  | *Breastfeeding* | Breastfeeding also part of antibiotic propensity score, which was adjusted for |  |
|  |  |  |  |  |  |  | *Childhood infections* | Infections part of antibiotic propensity score, which was adjusted for |  |
