# Supplementary material for: The relationship of prenatal antibiotic exposure and infant antibiotic administration with childhood allergies: a systematic review
Source: BMC Pediatr. 2020 Jun 27;20:312. doi: 10.1186/s12887-020-02042-8 (PMC7320596; doi:10.1186/s12887-020-02042-8)

**Search strategy and process**

|  | **Pubmed** | **Embase** |
| --- | --- | --- |
| *Population* | ("Fetus"[Mesh] OR "Pregnancy"[Mesh:NoExp] OR "Maternal-Fetal Exchange"[Mesh] OR "Labor, Obstetric"[Mesh] OR fetus*[tiab] OR foetus*[tiab] OR fetal[tiab] OR foetal[tiab] OR maternal[tiab] OR perinatal[tiab] OR prenatal[tiab] OR peri-natal[tiab] OR pre-natal[tiab] OR intrapartum[tiab]) OR intra-partum[tiab]) OR delivery[tiab] OR early life[tiab] OR in utero[tiab] OR infant*[tiab] OR baby[tiab] OR babies[tiab] OR neonat*[tiab] OR newborn*[tiab] OR neonatal[tiab] OR postnatal[tiab] OR perinatal[tiab] OR prenatal[tiab] OR post-natal[tiab] OR peri-natal[tiab] OR pre-natal[tiab] OR "Infant"[Mesh] OR "Child"[Mesh] OR child[tiab] OR children [tiab]) AND | ('fetus'/exp OR 'fetus' OR 'pregnancy'/exp OR 'pregnancy' OR 'childbirth'/exp OR 'childbirth' OR fetus*:ab,ti,kw OR foetus*:ab,ti,kw OR fetal:ab,ti,kw OR foetal:ab,ti,kw OR maternal:ab,ti,kw OR pregnan*:ab,ti,kw OR intrapartum:ab,ti,kw OR delivery:ab,ti,kw OR 'in utero':ab,ti,kw OR infant*:ab,ti,kw OR baby:ab,ti,kw OR babies:ab,ti,kw OR children:ab,ti,kw OR 'child'/exp OR 'child' OR 'infant'/exp OR 'infant' OR 'baby'/exp OR 'baby' OR infancy:ab,ti,kw OR 'early life exposure'/exp OR 'early life exposure' OR 'early life'/exp OR 'early life' OR 'infancy'/exp OR 'infancy' OR neonat*:ab,ti,kw OR newborn*:ab,ti,kw OR neonatal:ab,ti,kw OR postnatal:ab,ti,kw OR perinatal:ab,ti,kw OR prenatal:ab,ti,kw OR 'post-natal':ab,ti,kw OR 'peri-natal':ab,ti,kw OR 'pre-natal':ab,ti,kw) AND |
| *Exposure* | ("Anti-Bacterial Agents"[Mesh] OR "Anti-Bacterial Agents" [Pharmacological Action] OR antibacterial*[tiab] OR anti- bacterial*[tiab] OR antibiotic*[tiab] OR antimicrobial[tiab] OR anti-microbial[tiab] OR anti-infective[tiab] OR antiinfective[tiab]) AND | ('antibiotic agent'/exp OR 'antibiotic agent' OR antibacterial*:ab,ti,kw OR 'anti-bacterial*':ab,ti,kw OR antibiotic*:ab,ti,kw OR antimicrobial:ab,ti,kw OR 'anti-microbial':ab,ti,kw OR 'anti-infective':ab,ti,kw OR antiinfective:ab,ti,kw) AND |
| *Outcome* | ("Asthma"[Mesh] OR "Rhinitis, Allergic"[Mesh] OR "Eczema"[Mesh] OR "Dermatitis, Atopic"[Mesh] OR (asthma [tiab] OR allergy[tiab] OR hay fever[tiab] OR allergic rhinitis[tiab] OR eczema[tiab] OR atopic dermatitis[tiab] OR atopic disease[tiab] OR atopy[tiab]) | ('asthma' OR 'asthma'/exp OR asthma OR 'atopic dermatitis'/exp OR 'atopic dermatitis' OR 'eczema' OR 'eczema'/exp OR eczema OR 'allergic rhinitis'/exp OR 'allergic rhinitis' OR 'pollen allergy'/exp OR 'pollen allergy' OR 'hay fever'/exp OR 'hay fever' OR 'allergy' OR 'allergy'/exp OR allergy) |
| *Filters* | period 01/01/2008 to 01/08/2018 | Filters: articles or ‘articles in press’ and period 01/01/2008 to 01/08/2018 |

**Table S1**. Search terms used to retrieve articles in Pubmed and Embase about prenatal and infant antibiotic exposure and the outcomes childhood asthma, eczema and hay fever

**Figure S1.** Search process for publications examining prenatal

or infant antibiotic exposure and childhood allergies


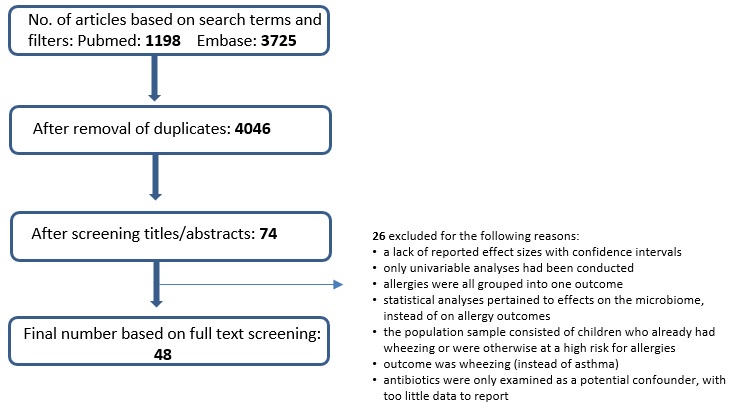

Supplement: Supplementary file 2 — Additional file 2. Supplementary data_search strategy and process: This file has a table containing the search terms and strategy used for Embase and Pubmed to retrieve relevant publications (Table S1) and a flowchart depicting the search process (Figure S1). [file 12887_2020_2042_MOESM2_ESM.docx]
